# QTL detection for growth and latex production in a full-sib rubber tree population cultivated under suboptimal climate conditions

**DOI:** 10.1186/s12870-018-1450-y

**Published:** 2018-10-10

**Authors:** João Ricardo Bachega Feijó Rosa, Camila Campos Mantello, Dominique Garcia, Lívia Moura de Souza, Carla Cristina da Silva, Rodrigo Gazaffi, Cícero Casimiro da Silva, Guilherme Toledo-Silva, Philippe Cubry, Antonio Augusto Franco Garcia, Anete Pereira de Souza, Vincent Le Guen

**Affiliations:** 1 Department of Genetics, Luiz de Queiros College of Agriculture (ESALQ), University of São Paulo (USP), Avenida Pádua Dias, 11, Pircacicaba, SP 13400-970 Brazil; 2 FTS Sementes S.A., Avenida Newton Slaviero, Ponta Grossa, PR 84043-560 Brazil; 3 Molecular Biology and Genetic Engineering Center (CBMEG), University of Campinas (UNICAMP), Campinas, SP Brazil; 4 National Institute of Agricultural Botany (NIAB), Huntingdon Road, Cambridge, CB3 0 LE UK; 5 CIRAD, UMR AGAP, F-34398 Montpellier, France; 6 AGAP, Univ Montpellier, CIRAD, INRA, INRIA, Montpellier SupAgro, Montpellier, France; 7 Center of Agronomic Sciences, Department of Biotechnology and Vegetal and Animal Production, Federal University of São Carlos (UFSCAR), Jardim Residencial Pedras Preciosas, Araras, SP 13604900 Brazil; 8 Plantation E. Michelin, R&D Department, Rua João de Barro quadra 22 lote 16, Ouro Branco do Sul, Itiquira, MT 78790-000 Brazil; 9 Laboratory of Biomarkers of Aquatic Contamination and Immunochemistry - LABCAI, Biochemistry Department, Federal University Santa Catarina, Florianópolis, Brazil; 10 IRD, UMR DiADE, 911 avenue Agropolis, BP 64501, 34394, Montpellier cedex 5, France; 11 Department of Plant Biology, Biology Institute, University of Campinas (UNICAMP), Campinas, SP Brazil

**Keywords:** *Hevea brasiliensis*, Rubber production, Mixed model, SNP marker, Genetic mapping, Marker-assisted selection

## Abstract

**Background:**

Rubber tree is cultivated in mainly Southeast Asia and is by far the most significant source of natural rubber production worldwide. However, the genetic architecture underlying the primary agronomic traits of this crop has not been widely characterized. This study aimed to identify quantitative trait loci (QTLs) associated with growth and latex production using a biparental population established in suboptimal growth conditions in Brazil.

**Results:**

A full-sib population composed of 251 individuals was developed from crossing two high-producing Asiatic rubber tree cultivars, PR 255 and PB 217. This mapping population was genotyped with microsatellite markers from enriched genomic libraries or transcriptome datasets and single-nucleotide polymorphism (SNP) markers, leading to construction of a saturated multipoint integrated genetic map containing 354 microsatellite and 151 SNP markers. Height and circumference measurements repeated over a six-year period and registration of cumulative latex production during six consecutive months on the same individuals allowed in-depth characterization of the genetic values of several growth traits and precocious latex production. Growth traits, circumference and height, were overall positively correlated, whereas latex production was not correlated or even negatively correlated with growth traits. A total of 86 distinct QTLs were identified, most of which were detected for only one trait. Among these QTLs, 15 were linked to more than one phenotypic trait (up to 4 traits simultaneously). Latex production and circumference increments during the last wintering period were associated with the highest numbers of identified QTLs (eleven and nine, respectively), jointly explaining the most significantly observed phenotypic variances (44.1% and 44.4%, respectively). The most important QTL for latex production, located on linkage group 16, had an additive effect of the male parent PB 217 and corresponded to a QTL at the same position detected in a previous study carried out in Thailand for the biparental population RRIM 600 x PB 217.

**Conclusions:**

Our results identified a set of significant QTLs for rubber tree, showing that the performance of modern Asiatic cultivars can still be improved and paving the way for further marker-assisted selection, which could accelerate breeding programs.

**Electronic supplementary material:**

The online version of this article (10.1186/s12870-018-1450-y) contains supplementary material, which is available to authorized users.

## Background

Although originating from South America, rubber tree (*Hevea brasiliensis*) is grown in mainly Southeast Asia and West Africa, wherein optimal edaphoclimatic conditions are reached to provide high rubber production. However, rubber tree may also be cultivated under suboptimal climate conditions when competing with essential food crops or when a disease threatens its durability in more favorable areas. The latter occurs in Brazil with South American leaf blight (SALB) disease, caused by the fungus *Microcyclus ulei* that is prevalent throughout most of the country. SALB has led rubber tree growers to concentrate their production in the State of São Paulo, a southeastern region of Brazil, where climatic conditions are less favorable for the epidemic development of SALB [1].

Rubber tree is cultivated mainly for the production of natural rubber, an indispensable commodity in all the aspects of modern daily life, mainly in the tire industry. Latex is regularly harvested by tapping the bark and is then transformed into standardized rubber after coagulation and various industrial processes, including cleaning, drying and pressing [2].

Compared with most food crops, the domestication history of rubber tree is very recent, initiating approximately 140 years ago. Until the beginning of the twentieth century, latex was collected only from wild rubber trees in the Amazonian forest and rudimentarily processed to produce balls of rubber [2], most of which were exported to Great Britain. In 1876, approximately 70,000 rubber seeds were collected in the Brazilian State of Pará by Henry Wickham and shipped to the Kew Botanic Gardens [3]. Because of a very low germination rate, only a few plantlets were dispatched from Kew Botanic Gardens to the Colombo Botanic Gardens in Sri Lanka and Singapore, where they were grown into adults, further multiplied and disseminated by seeds, giving rise to the genetic basis of rubber tree breeding. The first large-scale rubber tree plantations were established in Southeast Asia only a century ago [3] and originated from the few “Wickham” trees.

Initial rubber breeding strategies aimed at crossing the most vigorous and best latex yielding trees to produce selected seedlings for fast-growing areas on rubber tree plantations. When budding techniques were widely adopted in the 1920s [4], mass selection over many trees allowed the identification and multiplication of the first elite clones, which were also used as progenitors in future breeding programs, allowing noticeable progress in latex production [5]. Until now, this process has continued at a slow pace due to the lengthy period required to assess the genetic value of the breeding units. For that reason, modern rubber tree cultivars (clones propagated by budding) are distant from the first “Wickham trees” by only a few generations, probably no more than four or five in most cases [6]. Consequently, a small number of recombination/selection events should have occurred after introduction of the wild Wickham genotypes, and modern cultivars thus continue to carry unfavorable alleles at major genes encoding agronomic traits of interest that have not yet been eliminated via the selection process [7]. Genetic progress should therefore continue to be significant for an extended period by progressively alleviating this genetic load, and marker-assisted selection (MAS) should greatly improve the pace of genetic breeding and reduce the time required to develop new varieties [6].

To date, various genetic mapping studies have been performed to identify quantitative trait loci (QTLs) useful for MAS in rubber tree that are essentially focused on disease resistance. The first QTLs detected for rubber tree were associated with resistance to SALB after inoculation of the fungus *M. ulei* onto their leaves under controlled conditions [8]. Afterwards, this study was completed by others on the same genetic background but in different infestation conditions [9, 10] and was used to characterize QTLs arising from other resistant genotypes [11, 12]. All these results provide an overview of the genetic diversity associated with SALB resistance and the genomic complexity of rubber tree regarding the number of genetic factors and interactions with fungus isolates.

QTLs for other important agronomic traits, such as growth and latex production, were also identified in rubber tree. Rattanawong et al. [13] developed a full-sib population (196 individuals) derived from a cross between two “Wickham” clones (RRIM 600 x PB 217) and identified QTLs that explained high proportions (31 and 66%) of the observed phenotypic variances (*R*^*2*^) for both traits, respectively. Souza et al. [14] identified QTLs for two growth traits (height and circumference) in rubber tree based on a full-sib population (PR 255 x PB 217) evaluated in a Brazilian suboptimal climatic condition (hot and wet summer and an extended dry and relatively cold winter) for three consecutive years. The authors evaluated phenotypic data, separating data into summer and winter seasons, and mapped QTLs for each season and both seasons simultaneously. A total of 11 and 7 QTLs were detected for height and circumference growth traits, respectively. A lower proportion of observed phenotypic variance was observed for height (from 2.7 to 8.1%) and circumference (from 2.8 to 9.0%) compared to the estimated proportion of 31% previously detected by Rattanawong et al. [13]. Therefore, better understanding the genetic architecture of growth traits in different climatic conditions is necessary.

Confirming whether QTLs detected in Thailand for a specific population, cultivated under optimal climatic conditions [13], are the same as those identified in Brazil for another population under sub-optimal climatic conditions was of interest [14]. Although both studies used different segregating populations, they shared a parental clone (PB 217), which could be used for this confirmation. Comparative mapping between the studies by Rattanawong et al. [13] and Souza et al. [14] should be performed for only growth traits because the latter did not map QTLs for latex production in rubber tree. However, more recently, a high density SNP-based genetic map allowed the identification of 17 QTLs involved in the control of dry latex production [15].

Our objectives in the present study were the mapping of QTLs related to growth and latex production traits on rubber tree cultivated under the sub-optimal climatic conditions of Southeast Brazil. We have used for this purpose an experimental design that has already been described in a previous study [14], but without latex production until now because the trees were too young. We extended the previous multipoint genetic linkage map to include novel simple sequence repeat (SSR) and single-nucleotide polymorphism (SNP) markers. We used a linear mixed model approach to analyze a new growth (height and circumference) and latex production dataset. With two more years of growth data registration, most trees in the experimental field had reached a sufficient circumference to be tapped, providing latex production data. Finally, QTL analyses of growth and latex production traits were performed, advancing our understanding of their genetic architecture in rubber tree.

## Results

### Molecular marker genotyping

#### SSRs

Of the 364 SSRs initially available, 112 (30.8%) were polymorphic for the parent PR 255, 72 (19.8%) were polymorphic for the parent PB 217, and 178 (48.9%) were polymorphic for both parents. For the latter, 145 (81.5%) and 33 (18.5%) presented segregation ratios of 1:1:1:1 (fully informative) and 1:2:1, respectively.

From the 12 (2.2%) molecular markers that deviated from Mendelian segregation, following the Bonferroni correction for multiple tests, 10 (83.3%) comprised SSRs. Of these, five SSRs (50.0%, *A2746*, *t283*, *a268*, *TA2158* and *HBE64*), one (10.0%, *HB152*), and four (40.0%, *A2481*, *TAs2196*, *TAs2746* and *A2387*) deviated from the expected 1:1:1:1, 1:2:1 and 1:1 segregations, respectively.

#### SNPs

A total of 243 SNPs previously detected by Mantello et al. [16] and Silva et al. [17] were genotyped by Sequenom MassARRAY iPLEX technology in the segregating population. Of these, 54 (22.2%) failed to amplify, 83 (34.2%) were monomorphic, and 106 (39.6%) were polymorphic for at least one parent (PR 255 or PB 217), enabling their use in genetic mapping (Table 1). However, one of the polymorphic SNPs (*Hb_seq_50*) presented an incompatible segregation pattern to an F1 population [18] and was excluded from the Sequenom SNP dataset, resulting in 105 (39.18%) useful SNPs. One SNP (*Hb_seq_38_2*) presented deviation from the expected segregation at 1:2:1.

**Table 1:** Origin and nature of the molecular markers used to construct the genetic linkage map

Initial name	Marker	Library Type	Sequencing method	Reference	Genotyping Technology	Genotyped markers	Mapped markers
a, A, T, TAs	SSR	Enriched genomic library	Sanger	Le Guen et al. (2011) [11]	LI-COR 4300 DNA Analyzer	184	177
HBE	SSR	EST library	Sanger	Feng et al. (2009) [50]	Silver staining on acrylamide gels	58	58
EHB	SSR	EST library	Sanger	Silva et al. (2014) [17]	LI-COR 4300, silver staining, Advance	50	49
HB	SSR	Enriched genomic library	Sanger	Souza et al. (2009) [62] Mantello et al. (2012) [63]	Silver staining on acrylamide gels	64	62
SSH	SSR	SSH libraries	Sanger	Cubry et al. (2014) [49]	ABI 3500 xL Genetic Analyzer	6	6
HV	SSR	GenBank Nucleotide	Sanger	Gouvêa et al. (2010) [51]	Silver staining on acrylamide gels	2	2
					*sub-total SSRs*	*364*	*354*
Hb_seq_	SNP	RNA-seq	GAIIx, Illumina	This paper	Mass Array Analyzer, Sequenom	105	72
sHbUNI	SNP	RNA-seq and genomic library	GAIIx, Illumina	Mantello et al. (2014) [16], Souza et al. (2016) [64]	Fluidigm, BioMark HD	21	19
sHbCIR	SNP	RNA-seq	454-Roche	Salgado et al. (2014) [22]	Fluidigm, BioMark HD	65	60
					sub-total SNPs	191	151
					Total	555	505

In addition, a total of 96 SNPs (74 and 22, named *sHbCIR* and *sHbUNI*, respectively) were used to genotype the segregating population with Fluidigm® technology. From these, seven (7.3%, 6 *sHbCIR* and 1 *sHbUNI*) did not amplify, and three (3.1%, *sHbCIR*) were monomorphic, which led to their exclusion from the total dataset. Moreover, one additional SNP (*sHbUNI0351_S*) was excluded because it presented incompatible segregation [18]. Therefore, a total of 85 (88.5%) SNPs, 65 (76.5%) with the initial *sHbCIR* and 20 (23.5%) with the initial *sHbUNI*, were available. Only the SNP *sHbUNI0515_S* deviated from the 1:2:1 segregation pattern.

### Integrated genetic map

**Fig. 1:**
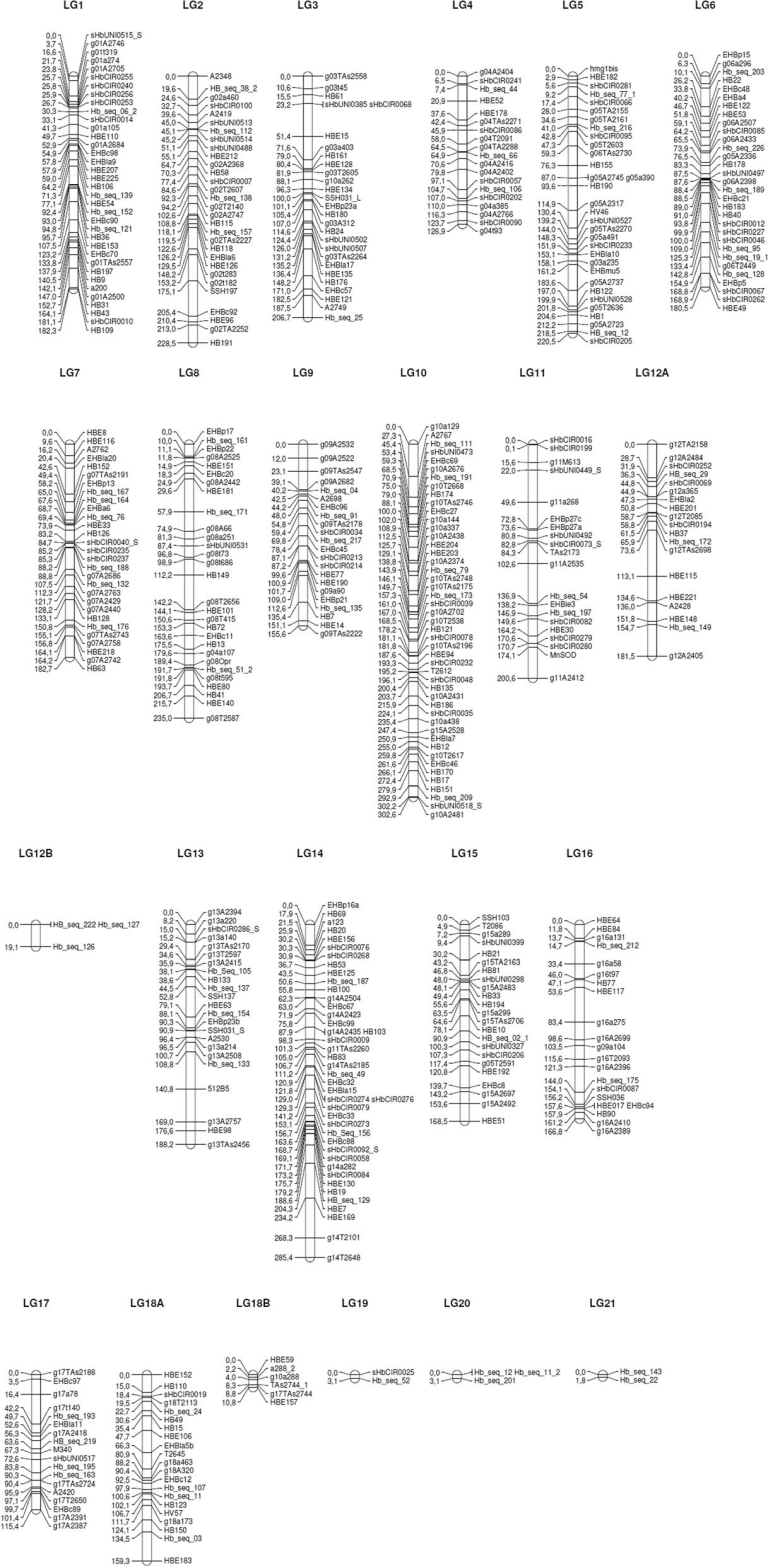
Integrated genetic linkage map for the rubber tree PR 255 x PB 217 population

By combining data from all the genotyping techniques, 555 markers, 364 SSRs and 191 SNPs, were used to construct the integrated genetic linkage map. From these, 354 SSRs and 151 SNPs were positioned into the final genetic linkage map (Fig. 1 and Additional file 1: Table S1). A total of 505 molecular markers were positioned on 23 linkage groups.

The largest of these 23 linkage groups was LG10 with a total of 47 (9.3%) molecular markers (35 SSRs and 12 SNPs) and an extension of more than 300 centiMorgan (cM) (8.6%). The smallest groups were LG19, LG20 and LG21 with no more than three (0.6%) SNP markers and 5 cM of extension. Two linkage groups, LG12B and LG18B, were considered part of LG12 and LG18, respectively, according to graphical analysis performed for each linkage group.

In general, the 151 SNP markers, which were developed and mapped for the first time in the present F1 population, were distributed to all the linkage groups except LG18B. The linkage groups comprising the most SNPs were LG14 (14 SNPs), LG6 (13 SNPs), LG10 (12 SNPs), LG1 (11 SNPs), and LG5 (10 SNPs) and LG11 (10 SNPs).

### Phenotypic traits

Several variance-covariance structures for the genetic and residual matrices investigated and compared via Akaike information criteria (AIC) are summarized in Additional file 1: Tables S2 and S3, respectively. The selected models (Additional file 1: Table S4) indicated heterogeneity of genetic variance for height (SH2, WH1, WH2, and TWH), circumference (SC1, SC4, SC5, WC2, WC3, and WC4), and total latex production (TLP) with the structures DIAG, FA, and AR1_Het_. Genetic correlations were also observed for growth traits and latex production with the models FA and AR1_Het_. Moreover, heterogeneity of residual variance (SH1, TSH, TWH, TSWH, SC1, SC4, SC5, WC1, WC2, WC3, WC4, and TLP) and presence of residual or spatial correlation (TSH, WH1, SC1, SC3, SC4, SC5, TSC, WC3, WC4, TWC, TSWC, and TLP) were detected with the models DIAG, AR1, and AR1_Het_.

**Fig. 2:**
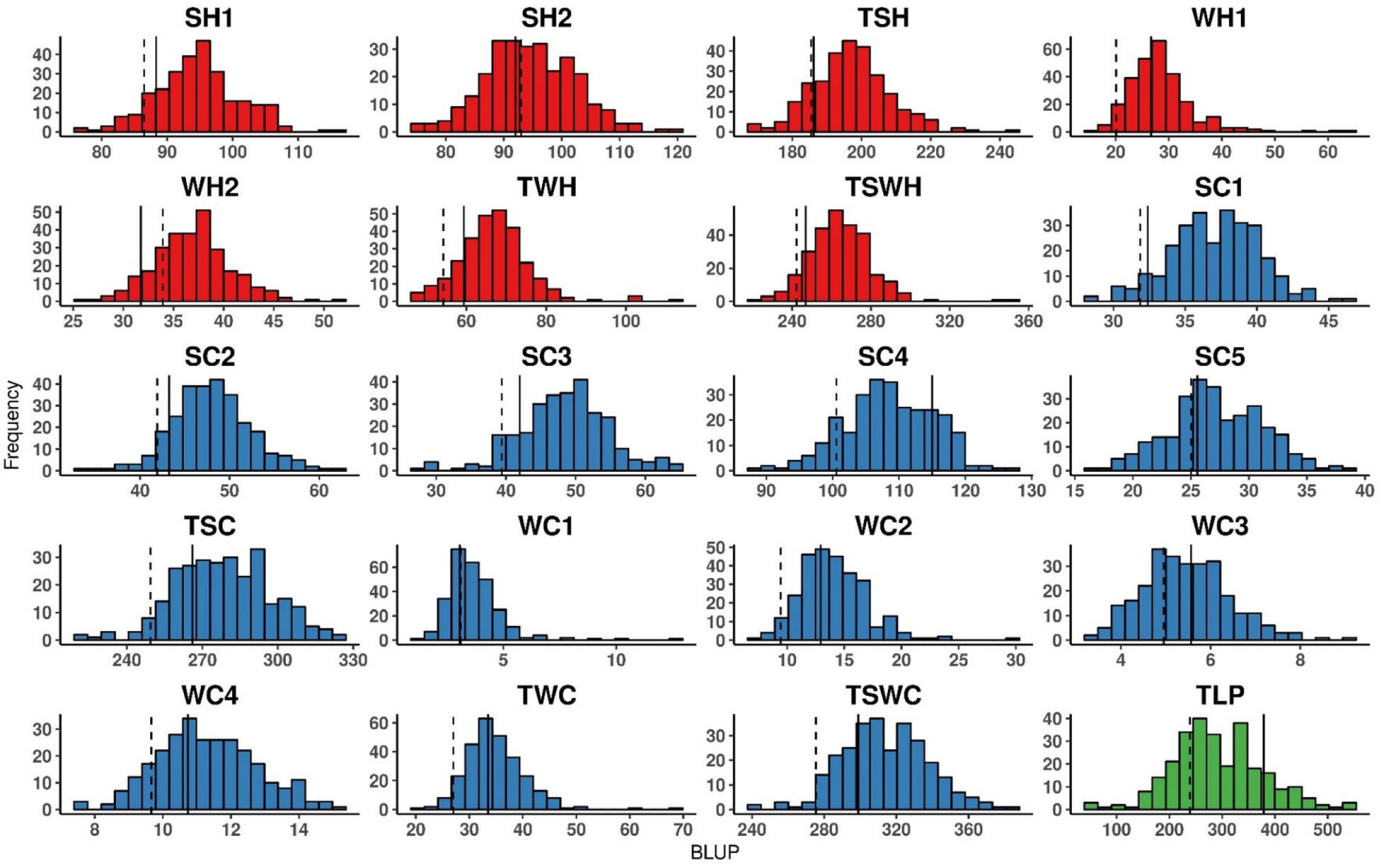
Predicted genetic values of the traits. Distribution of the predicted genetic values (BLUPs) of the PR 255 x PB 217 population, obtained from the phenotypic analyses via mixed models, for the traits related to height, circumference, and latex production. Histograms of traits related to height, circumference, and latex production are indicated with colours red, blue, and green, respectively. BLUPs of the parents PR 255 and PB 217 are vertically presented in each histogram with continuous and dotted lines, respectively. SH: Summer Height; WH: Winter Height; TSH: Total Summer Height; TWH: Total Winter Height; TSWH: Total Summer Winter Height; SC: Summer Circumference; WC: Winter Circumference; TSC: Total Summer Circumference; TWC: Total Winter Circumference; TSWC: Total Summer Winter Circumference; TLP: Total Latex Production. The number in the end of some variable names indicates the year of measurement repeated in time for height and circumference

Table 2 and Fig. 2 show the main features and distributions of the 20 variables that were analyzed from height, circumference, and latex production measurements. Comparing the predicted genotypic values of the mapping population parents, PR 255 was slightly superior to PB 217 for all growth variables and markedly superior for TLP. Moreover, the predicted genotypic values of the two parents regarding height and circumference for both summer and winter seasons increased over time. The predicted genotypic values of the mapping population were always higher for the summer season than the winter season, and this superiority was evident for both height and circumference traits (Table 2 and Fig. 2).

**Table 2:** Main characteristics related to height, circumference, and latex production traits

Trait	Parentage		Population		Variances, Heritability, and Coefficient of Variation				
	PR 255	PB 217	Average	Range	${\sigma}_G^2$	${\sigma}_e^2$	${\sigma}_t^2$	*h* ^2^	*CV*%
SH1	88.3	86.5	94.9	75.9–115.3	57.3	188.9	304.7	0.19	14.5
SH2	92.0	93.0	94.6	74.3–118.9	70.7	175.1	245.8	0.29	14.0
TSH	186.2	185.4	197.3	170.1–244.8	162.5	668.9	1066.5	0.15	13.1
WH1	26.7	20.1	28.4	15.4–64.0	27.5	40.1	67.6	0.41	22.3
WH2	31.7	33.9	36.9	25.9–51.7	23.4	93.1	138.4	0.17	26.2
TWH	59.3	54.2	67.0	48.2–113.0	73.5	152.2	242.6	0.30	18.4
TSWH	246.9	242.3	264.5	223.2–354.4	349.5	750.1	1303.6	0.27	10.4
SC1	32.4	31.9	37.2	28.0–46.0	12.0	32.2	56.7	0.21	15.3
SC2	43.3	41.9	48.2	33.6–62.5	23.8	30.6	67.5	0.35	11.5
SC3	42.0	39.4	48.5	28.1–65.1	49.5	41.6	105.0	0.47	13.3
SC4	115.0	100.6	108.6	87.2–126.1	60.3	108.4	192.1	0.31	9.6
SC5	25.6	25.0	27.2	16.7–38.9	0.0	16.1	19.9	0.00	14.8
TSC	266.1	249.4	278.8	221.0–323.8	478.3	507.9	1028.4	0.47	8.1
WC1	3.1	3.1	3.7	1.1–12.6	2.0	2.4	4.4	0.45	41.6
WC2	12.9	9.4	14.1	8.6–29.8	10.1	9.6	22.3	0.45	22.2
WC3	5.6	5.0	5.5	3.3–9.1	1.4	3.6	5.3	0.26	34.8
WC4	10.7	9.7	11.3	7.5–15.1	0.9	4.3	6.1	0.14	18.4
TWC	33.5	27.0	35.0	21.1–69.4	36.8	25.9	70.9	0.52	14.6
TSWC	298.9	275.4	314.3	241.4–384.7	700.6	606.0	1389.9	0.50	7.9
TLP	378.7	239.0	291.6	60.7–550.4	2447.5	1002.7	3450.2	0.71	10.8

Broad-sense heritabilities ranged from 0.15 to 0.41 for height traits, from 0.00 to 0.52 for circumference traits, and reached 0.71 for TLP (Table 2). Therefore, higher participation of the detected QTLs was expected for the observed phenotypic variance for latex production than that for circumference and even more than for height.

Genetic correlations between growth traits, both height and circumference, were overall positive and statistically significant at 0.1, 1, and 5% (Table 3). TLP was not correlated with most growth traits, and when correlations were observed, they were always negative with weak (SH1), moderate (WH1, TWH, TSWH, WC1), and high (SC5) Pearson’s coefficients. Interestingly, Pearson’s correlation coefficients varied within the circumference measurements. Genetic correlation decay over time is possible, showing that genotypic values from near measurements are more correlated than those from more distant measurements. For example, SC1 was more genetically correlated with SC2 (0.61) than SC3 (0.46), SC4 (0.28), and SC5 (− 0.06). The same growth trait evidence was observed for the winter season, as WC1 was more genetically correlated with WC2 (0.53) than WC3 (0.23) and WC4 (0.15).

**Table 3:** Pearson correlation coefficients between traits related to height, circumference, and latex production in the rubber tree population

Trait	SH2	TSH	WH1	WH2	TWH	TSWH	SC1	SC2	SC3	SC4	SC5	TSC	WC1	WC2	WC3	WC4	TWC	TSWC	TLP
SH1	0.08	0.70^*^	0.17^+^	0.15^▪^	0.17^+^	0.63^*^	0.32^*^	0.18^+^	0.17^+^	0.22^*^	0.12^▪^	0.24^*^	0.11	0.17^+^	0.07	0.20^*^	0.23^*^	0.26^*^	-0.16^+^
SH2		0.55^*^	0.09	-0.10	-0.04	0.47^*^	0.20^*^	0.38^*^	0.35^*^	0.06	-0.07	0.27^*^	0.25^*^	0.13^▪^	0.02	0.07	0.18^+^	0.27^*^	-0.06
TSH			0.15^▪^	0.07	0.10	0.83^*^	0.30^*^	0.32^*^	0.29^*^	0.14^▪^	0.02	0.27^*^	0.20^+^	0.19^+^	0.07	0.19^+^	0.26^*^	0.28^*^	-0.11
WH1				0.34^*^	0.76^*^	0.50^*^	0.21^*^	0.29^*^	0.24^*^	0.05	0.10	0.23^*^	0.47^*^	0.31^*^	0.09	0.11	0.36^*^	0.28^*^	-0.19^+^
WH2					0.80^*^	0.43^*^	0.15^▪^	0.19^+^	0.20^*^	0.19^+^	0.10	0.25^*^	0.25^*^	0.33^*^	0.05	0.11	0.31^*^	0.28^*^	-0.11
TWH						0.54^*^	0.23^*^	0.28^*^	0.26^*^	0.15^▪^	0.13^▪^	0.29^*^	0.39^*^	0.38^*^	0.09	0.13^▪^	0.38^*^	0.34^*^	-0.21^*^
TSWH							0.34^*^	0.41^*^	0.37^*^	0.20^*^	0.08	0.37^*^	0.36^*^	0.33^*^	0.11	0.21^*^	0.39^*^	0.40^*^	-0.18^+^
SC1								0.61^*^	0.46^*^	0.28^*^	-0.06	0.59^*^	0.17^+^	0.39^*^	0.15^▪^	0.08	0.33^*^	0.59^*^	-0.06
SC2									0.69^*^	0.48^*^	-0.10	0.75^*^	0.32^*^	0.43^*^	0.29^*^	0.19^+^	0.46^*^	0.76^*^	0.02
SC3										0.52^*^	0.09	0.79^*^	0.42^*^	0.59^*^	0.20^+^	0.34^*^	0.60^*^	0.81^*^	-0.01
SC4											0.39^*^	0.79^*^	0.04	0.33^*^	0.24^*^	0.45^*^	0.41^*^	0.76^*^	0.05
SC5												0.36^*^	-0.02	0.02	-0.03	0.37^*^	0.12	0.33^*^	-0.36^*^
TSC													0.27^*^	0.48^*^	0.24^*^	0.42^*^	0.55^*^	0.98^*^	-0.06
WC1														0.53^*^	0.23^*^	0.15^▪^	0.65^*^	0.37^*^	-0.18^+^
WC2															0.33^*^	0.21^*^	0.85^*^	0.61^*^	-0.07
WC3																0.28^*^	0.58^*^	0.33^*^	-0.05
WC4																	0.56^*^	0.48^*^	0.05
TWC																		0.69^*^	-0.09
TSWC																			-0.07

Pearson correlation coefficients were tested and declared as statistically significant using *t* test (^*^: *p* < 0.001; ^+^: *p* < 0.01; ^▪^: *p* < 0.05)

### QTL mapping

Unlike the previous study performed with the same F1 segregating population [14], in this work, QTLs were mapped for growth traits computed for all time intervals between the first and last measurements and for TLP. All genomic positions with LOD scores detected with composite interval mapping (CIM) methodology [19] that were superior to thresholds determined by permutation tests [20, 21] were considered possibly significant QTLs (Additional file 1: Table S5). Considering all 20 phenotypic traits, a total of 111 putative QTLs were detected based on LOD score thresholds obtained by the method established by Chen and Storey [21]. When a statistically significant locus appeared near another (distance smaller than 5 cM) for a different trait, we considered these loci a unique QTL. We were thus able to merge 47 of 111 LOD score peaks into 19 QTLs because of their genetic proximity on the genetic map, consolidating the number of QTLs to 83 (Additional file 1: Table S5).

The genetic architecture of the evaluated traits was dissected in depth for the rubber tree population (Additional file 1: Table S5), containing the number of QTLs and the theoretical participation of genetic factors to the observed phenotypic variance (*R*^*2*^) for all traits. Surprisingly, the growth QTLs detected in cumulative seasonal periods (TSH, TWH, TSC, and TWC) were different from those detected at seasonal intervals for the same trait, showing the importance of the strategy adopted in this work. However, some exceptions were observed, such as LG02–40 (SC2, SC3, SC4, and TSC), LG03–96/93 (SC3 and SC4), LG03–116 (WC1 and TWC), LG03–184 (SC3 and TSC), LG08–60 (WC3, WC4, and TWC), LG08–219 (SC2 and SC3), LG16–4 (SC2 and TSC), LG16–45 (SC3 and SC4), and LG15–100 (WC2 and TWC). According to the criterion of requiring a genetic distance less than 5 cM to declare a QTL unique, all of these cases represent unique QTLs for rubber tree circumference expressed over time.

**Fig. 3:**
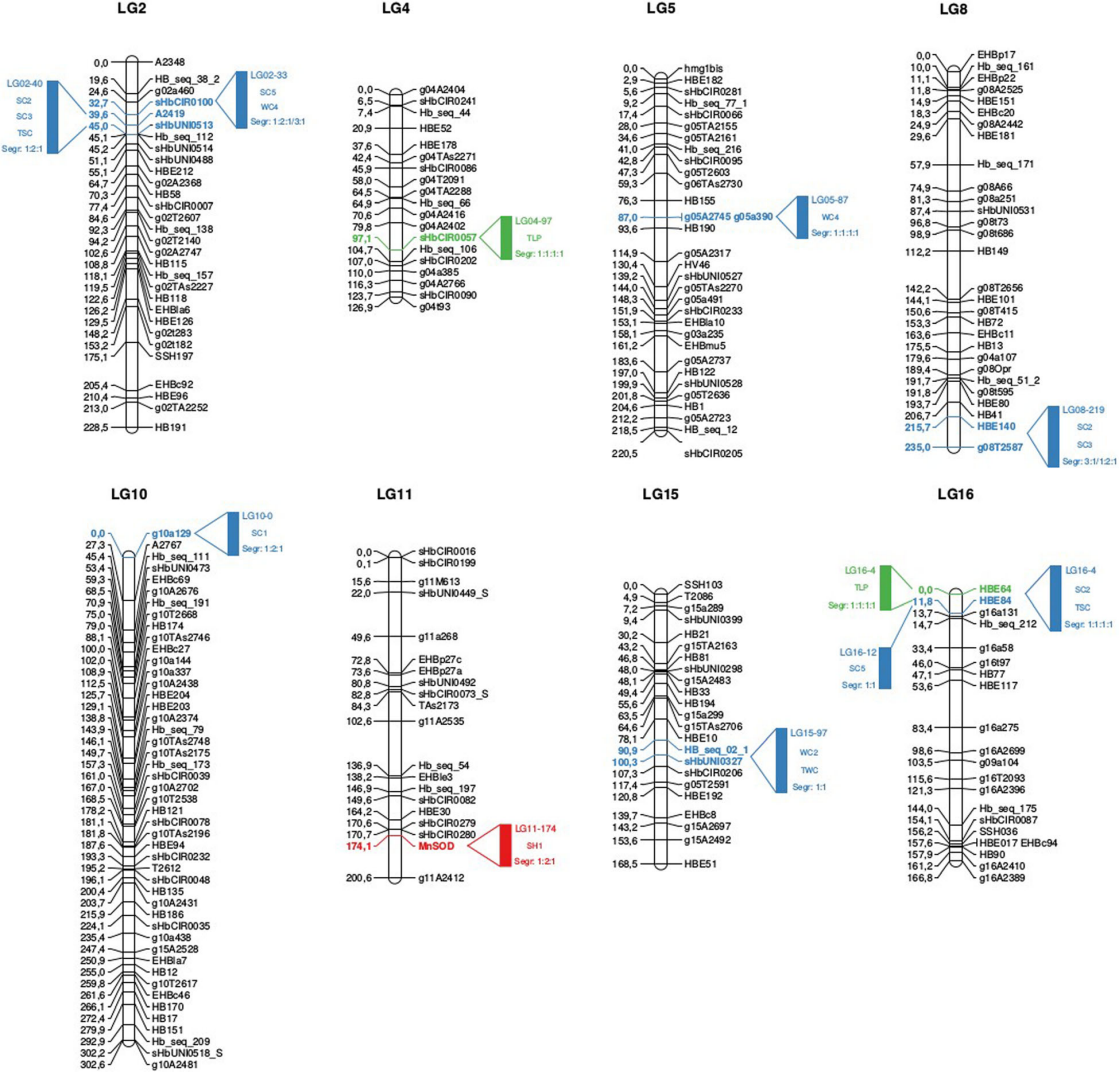
Location of 10 selected QTLs on the genetic map. Representation of 10 selected quantitative trait loci (QTLs) mapped to height, circumference, and latex production for an F1 population (PR 255 x PB 217) in the genetic linkage map. QTLs related to height, circumference, and latex production are indicated with colours red, blue, and green, respectively, containing the name (linkage group – LG merged with the estimated position in centimorgan), trait, and segregation. Flanking markers (right) and estimated position (left) are also indicated with the respective colours as described above

Table 4 and Fig. 3 list what we considered the 10 (11.6%) most important QTLs in the present study either because of their implication in several traits or because of their high contribution to the phenotypic variances of these traits. Out of these ten selected QTLs, four deserve to be highlighted.

**Table 4:** Characteristics of 10 selected QTLs mapped to height, circumference, and latex production for an F1 population (PR 255 x PB 217)

QTL name	Trait	Flanking Markers	Linkage Group (LG)	Position in cM	Global LOD	*R* ^*2*^	α_PR 255_ (LOD)	α_PB 217_ (LOD)	δ_PR 255 x PB 217_ (LOD)	Segregation
LG02–33	SC5	sHbCIR0100	2	32.70	4.623	6.877	−0.929 (3.038)	− 0.757 (2.564)	−0.314 (0.371)	1:2:1
	WC4	sHbCIR0100	2	32.70	8.636	7.221	−0.454 (4.998)	−0.388 (5.179)	− 0.242 (1.723)	3:1
LG02–40	SC2	A2419 – sHbUNI0513	2	43.00	5.125	5.567	−0.712 (1.802)	0.251 (0.228)	−0.989 (3.075)	1:2:1
	SC3	A2419	2	39.59	11.116	0.472	−2.102 (7.578)	0.236 (0.108)	−1.604 (4.336)	1:2:1
	TSC	A2419	2	39.59	17.732	14.426	−7.189 (11.365)	0.174 (0.007)	−6.086 (7.687)	1:2:1
LG04–97	TLP	sHbCIR0057	4	97.15	13.252	8.678	14.391 (2.077)	−32.543 (12.118)	8.657 (0.772)	1:1:1:1
LG05–87	WC4	g05a390	5	86.99	9.921	8.525	0.497 (8.207)	0.248 (2.537)	0.012 (0.004)	1:1:1:1
LG08–219	SC2	HBE140 – g08 T2587	8	219.00	3.625	3.941	−0.545 (0.956)	−0.782 (1.469)	−0.693 (0.979)	3:1
LG08–219	SC3	HBE140 – g08 T2587	8	219.00	6.848	7.267	0.431 (0.301)	−1.888 (4.226)	− 1.037 (1.194)	1:2:1
LG10–0	SC1	g10a129	10	0.00	3.623	6.449	0.579 (2.006)	−0.881 (1.811)	0.377 (0.376)	1:2:1
LG11–174	SH1	MnSOD	11	174.08	8.063	6.479	−1.390 (3.237)	−2.207 (5.511)	−0.609 (0.585)	1:2:1
LG15–97	WC2	sHbUNI0327	15	100.28	4.413	5.550	−0.745 (4.030)	0.190 (0.234)	−0.049 (0.016)	1:1
	TWC	HB_seq_02_1 – sHbUNI0327	15	97.00	6.842	6.746	−1.833 (5.119)	0.740 (0.574)	0.424 (0.239)	1:1
LG16–4	SC2	HBE64	16	0.00	6.720	3.375	0.039 (0.005)	1.792 (5.306)	0.690 (1.719)	1:1:1:1
	TSC	HBE64 – HBE84	16	4.00	5.137	3.932	−1.531 (0.420)	4.254 (3.789)	1.722 (0.515)	1:1:1:1
	TLP	HBE64 – HBE84	16	6.00	12.747	9.184	−3.824 (0.163)	−32.267 (10.682)	−18.152 (3.223)	1:1:1:1
LG16–12	SC5	HBE84	16	11.76	6.242	6.328	0.032 (0.005)	1.135 (5.931)	0.218 (0.218)	1:1

LG02–40 represents a QTL detected for circumference increments during the summer season on two intervals of measurement (SC2 and SC3) and the total circumference increment (TSC), with a maximum contribution to phenotypic variance (*R*^*2*^) of 14.4%. This QTL was due to the significant additive effect of the female parent PR 255 and the significant dominance effect from the interaction between the alleles of both parents for the three mentioned traits.

QTL LG05–87 contributed to the circumference increment during the fourth winter season (WC4). This QTL explained 8.5% of the phenotypic variation, also mainly due to the significant additive effect of the PR 255 parent, although the additive effect of the male parent PB 217 was also significant and contributed to the circumference increment.

LG16–4 is an important QTL detected for TLP, which contributed to 9.2% of the phenotypic variance. This QTL was also implicated in the SC2 and TSC traits and was very conditioned by the significant additive effect of the male parent PB 217 for the three traits. Moreover, a significant dominance effect from interaction between the PR 255 and PB 217 alleles was observed for SC2 and TLP.

LG04–97 is a QTL that also largely influenced TLP, as it contributed to 8.7% of the phenotypic variance due to significant additive effects of both parents. The segregations of this QTL and the other three described above were all 1:1:1:1.

## Discussion

The two primary objectives of any rubber tree breeding program are generally to (1) develop cultivars that grow rapidly and (2) produce high quantities of dry rubber. Therefore, identification of genetic factors that could help breeders reach these goals is of great importance, but few studies have focused on this topic. The field experimental design previously studied by Souza et al. [14] was performed ideally for the acquisition of growth (height and circumference) and production data as well as for the detection of QTLs. The recently acquired data from new growth measurements and from latex production through tapping provided an opportunity to follow the expression of growth-related QTLs over time and identify latex production-related QTLs in rubber trees cultivated under southeast Brazilian conditions. To our knowledge, this is the first report to map QTLs for growth and latex production traits in such conditions by exploring data collected over seven consecutive years.

One of two significant steps in the present QTL study was the establishment of an updated genetic linkage map previously constructed by Souza et al. [14]. In addition to SSR markers used in that work and novel SSR markers, the new genetic linkage map encompasses 151 SNP markers identified based on cDNA libraries obtained under varied conditions of identified tissues [16, 17, 22]. These SNP markers are derived from annotated sequences of various types of expressed genes and could thus be very useful in identifying possible genes underlying QTLs. Furthermore, these mapped SNPs will undoubtedly contribute to anchoring the recently released rubber tree genomes [23–25] to a genetic map, providing important advances for better representing the genome.

The high number of markers used to construct the new genetic linkage map contributed to both reducing the average interval between two adjacent markers (7.4 cM versus 9.9 cM) [14] and to increasing the total map length (3525 cM versus 2838 cM) [14]. Compared to other genetic linkage maps already published for rubber tree, the total lengths of both maps are higher, as the total lengths obtained on other populations were smaller (2144 cM [26], 2075 cM [27], 2441 cM [11], and 2094 cM [15]). Comparison of length for each linkage group was only possible with previous studies which have SSR markers in common with our work [11, 26, 27]. They show an overall good coherence in the relative lengths of their linkage groups. Conversely, comparisons were not possible with genetic maps constructed without any common marker with other studies [15]. The fact that the number of linkage groups in our study is superior to the haploid number of chromosomes (*n* = 18) in the rubber tree genome is probably because we have linkage groups with a small number of markers in some genomic regions, which will be linked with the others as we add more markers. However, the incorporation of more markers and their effective use for constructing an updated genetic linkage map will specifically depend on the genomic region and its recombination rate variation.

The breakdown of growth traits in elementary time interval increments facilitated the understanding of growth evolution during young stages (Table 2). Height growth was faster during the summer season than in winter and remained constant over the two summer intervals evaluated in time. The higher coefficient of variation for height in winter than in summer may be explained by the fact that the terminal apex was more subjected to biotic (pests) as well as abiotic (chilling temperatures) stresses during winter, which could heavily disrupt height growth and increase residual errors. This phenomenon may also affect the heritability and power to detect QTLs.

Circumference increments during summer increased from the first to the fourth interval but did not increase during the winter, instead remaining at a very low level. Consequently, the total circumference increment was highly correlated to the summer increment and its elementary intervals and less intensively correlated with the increment during winter. The circumference increment during the last summer interval of measurement (SC5) showed somewhat different behavior because it corresponded to the increment during latex production. Competition should exist for allocating photoassimilates between vegetative growth and regeneration of rubber particles in latex [28], which explains the very low level and reduced genetic variability of SC5 and its absence of correlation with most other growth traits. For this same reason, TLP was significantly negatively correlated with SC5 and showed no positive correlation with other growth traits.

A temporal decay of genetic correlation was observed for circumference in both summer and winter seasons (Table 3). This behavior is expected in semi-perennial or perennial crops repeatedly evaluated over time, such as sugarcane [29, 30], rubber tree (in the present study), eucalyptus [31], and pinus [32], and occurs because near measurements tend to be more genetically correlated than distant measurements. In rubber tree, measurements performed in the first years usually present lower circumferences compared to those carried out in subsequent years. Thus, genetic correlation between the first two measurements will naturally be higher than that between any of the more advanced measurements.

A total of 83 different QTLs were identified herein, and the genetic architecture of the traits was established based on the theoretical values of genetic participation in the phenotypic variance, the estimated effects, and the segregation of these QTLs (Additional file 1: Table S5). Most of the growth trait-related QTLs were detected only once in a specific elementary time interval, which indicates that they corresponded to specifically expressed genes or genetic factors during a determined period of development. Furthermore, with rare exceptions, QTLs detected in total periods did not correspond to those detected during the time intervals for the same traits. In other words, we could establish a distinction between “temporary QTLs” with a sufficient influence on growth traits in a limited period but without a long-term effect and “permanent QTLs” with a possible detectable effect on time intervals and significant cumulative effects when considering variation across several intervals. Finally, few QTLs were common for the same growth trait during both the summer and winter seasons or for height and circumference increments simultaneously. Only one QTL detected for TLP was also observed for circumference traits (SC2 and TSC).

Complex agronomic traits, such as growth, are usually controlled by many genes, which may explain the high number of QTLs associated with these traits that are usually detected in perennial plants. More than 60 different QTLs were found by Segura et al. [33] for the architecture of branching in apple trees. In a *Populus* interspecific family, a total of 82 QTLs were identified for growth based on genotype-environment (G x E) interactions [34]. In *Eucalyptus globulus*, 98 QTLs were identified for wood properties and growth [35].

However, if the identification of numerous QTLs helps to understand the genetic architecture of these important traits, they cannot all be used by breeders. Three major constraints shape the breeding schemes of rubber tree: (1) low rate of female fertility, which hampers the achievement of crosses with numerous genotypes; (2) long duration required to assess phenotypic performances (currently from 12 to 15 years to have an acceptable evaluation of rubber production); and (3) long duration of the biological cycle, a minimum of 6 years between one seed and the seed of the next generation. For these reasons, trying to gather favorable alleles for all QTLs by multiplying the generations is not reasonable. Furthermore, being a recently domesticated plant, rubber tree can still be greatly improved by QTLs of large effects. In that way, QTL LG16–4, whose position is between 0 and 6 cM on LG16, explains 9.2% of TLP phenotypic variance (and is also involved in SC2 and TSC), is an excellent MAS program candidate for rubber tree. The effect of this QTL is predominantly due to the additive effect of the male parent PB 217 (Table 4 and Additional file 1: Table S5) and corresponds precisely to the QTL identified by Rattanawong et al. [27] located on the same linkage group 16 at 5.8 cM, with R^2^ varying from 20 to 59%. In this latter case, the effect is due to the additive effects of both Asiatic parents (RRIM 600 and PB 217). Our work thus confirms the importance of this QTL identified in Thailand and its efficiency in different cultivation conditions.

Furthermore, the marker HBE64, mapped at 4.0 cM from this QTL, and the only marker mapped in this interval, had the best blast hit with the sieve element occlusion protein. Recent studies in rubber tree revealed that this protein causes laticifer plugging, as it may assist in the aggregation of rubber particles. Moreover, abundances of both the sieve element occlusion protein and its mRNA were reduced after etephon treatment, which facilitated and may have a direct relationship with latex flow [36]. Although different versions of the rubber tree genome have recently been published [23–25], a genome annotation is not publicly available. Thus, this does not allow an accurate investigation of genes in this region to more precisely delimitate the region that controls production. These results also show that although RRIM 600 and PR 255 are half-sib genotypes (the former from the cross TJIR 1 × PB 86 and the latter from the cross TJIR 1 × PR 107), they do not share the same favorable latex production allele.

In addition to LG16–4, an efficient MAS based on the family studied here (PR 255 × PB 217), should consider the QTL LG04–97, which also presents significant additive effects from PB 217 and PR 255 on latex production. The marker sHbCIR0057, mapped to these QTLs, had the best blast hit with sucrose synthase 4 (HbSus4). The activity of sucrose synthase was reportedly reduced due to treatment of the bark with Ethrel (2–chloroethylphosponic acid, an ethylene releaser) [37]. Previous studies on rubber tree demonstrated significant repression of HbSus4 under Ethrel treatment, which is consistent with the weakened enzymatic activity of Sus proteins in latex after Ethrel treatment [38]. These two candidate genes – sieve element occlusion protein and sucrose synthase – were not identified in the recent study of Xia et al. [15], and can therefore be added to the list of genes whose polymorphism can partially explain variations of latex production. Furthermore, the QTL LG02–40 seems to have a determinant influence on circumference increments with a significant additive effect from the parent PR 255 and a significant dominance effect between the alleles of both parents (Table 4 and Additional file 1: Table S5).

These findings are very interesting because they demonstrate that even if the genetic basis of Asiatic rubber clones is narrow due to their common origin from the first Wickham introduction, many favorable alleles for important agronomic traits are not yet fixed by selection in the “Wickham” population or shared by all rubber tree cultivars. Therefore, an opportunity still exists for further genetically improving the “Wickham” population by trying to concentrate favorable additive effects in the same genotypes.

## Conclusions

In conclusion, the rapid changes in genotyping technologies provide an excellent opportunity to improve the efficiency of MAS even for specifically difficult plants, such as rubber tree. Genotyping by sequencing should efficiently and quickly provide numerous SNP markers on every biparental population used in breeding schemes and on every unstructured population containing accessions from germplasms constructed to assess genetic diversity. These potentially numerous markers combined with rather good heritabilities for the most important traits (0.50 for TSWC and 0.70 for TLP) and limited numbers of QTLs for those same traits (8 QTLs for TSC and 11 for TLP) should be favorable for genome-wide selection (GWS) for the former population and genome-wide association studies (GWAS) for the latter population. In the first case, simulations carried out by Combs and Bernardo [39] showed that the accuracy of GWS under these conditions should be sufficient to shorten the duration of one breeding cycle by avoiding systematic phenotypic observations. As already demonstrated on oil palm, another perennial crop [40], we can hypothesize that an accurate estimation of genomic breeding value, carried out by simultaneous phenotyping and genotyping a training population, would allow a more efficient preselection in a large population of candidate genotypes and eventually a greater genetic gain per time unit on key agronomic traits like growth or latex production.

## Methods

### Plant material

An F1 (full-sib) segregating population was obtained by crossing the clone PR 255 (TJIR 1 x PR 107), the female parent, and the clone PB 217 (PB 5/51 x PB 6/9), the male parent, generating a total of 270 individuals (progenies). The seedlings obtained under controlled pollination were grown in a plastic bag nursery and then moved to the field before being clonally propagated by budding onto rootstocks.

Because both parental clones are modern Asiatic rubber tree cultivars, the clones originated from the first “Wickham trees”. PR 255 is a vigorous and high-yielding quick-starter clone [41] and was recommended for planting in Malaysia during the 1980–1994 period [42]. Under the edaphoclimatic conditions of southeast of Brazil, PR 255 is a rather good clone for growth and latex production stability [43]. PB 217 is a slow-growing cultivar during the early stages of its development and a slow-starter latex producer, meaning that its latex production increases progressively during the first years. However, this cultivar is well appreciated by rubber producers for its exceptional yield potential over a long-term period. This feature is mainly due to the high sucrose content of its latex, allowing intense hormonal stimulation and tapping [44], and this clone was also recommended in Malaysia from 1980 to 1994.

The two parental clones originated from Indonesia (PR255) and Malaysia (PB217) but were largely widespread throughout all rubber tree cultivating countries many years before the Convention on Biological Diversity (Rio de Janeiro, 1992). For that reason, they can be freely planted for commercial purposes, and used as progenitors in breeding and research programs. Controlled pollinations were carried out on the Edouard Michelin Plantation (Brazil), a Company that has been recognized as plant breeder by the Brazilian Ministry of Environment (CGEN, Decision n° 415, February 18th 2014). Michelin Company is therefore authorized to create new plant material by crossing and to study it for breeding purposes.

### Field trial

Progeny individuals of the F1 segregating population were multiplied by grafting onto rootstocks and planted in a field trial in Itiquira, Mato Grosso State, Brazil (17°24′03” S and 54°44′53” W) from March 2006 to March 2007. The climatic conditions of this region, characterized by a hot and wet summer and an exhaustively dry and relatively cold winter, are similar to those of southeast Brazil, which is the central Brazilian region for rubber tree cultivation. The experimental design comprised a randomized complete block design with four replicates and four grafted trees of the same individual per elementary plot. Therefore, each block consisted of 272 elementary plots, one plot for each of the 270 F1 individuals and one plot for each of the parental clones PR 255 and PB 217.

### Phenotypic measurements

On rubber plantations, trees are considered “young” or “immature” as long as they are not tapped for latex production, which occurs between six and 9 years old according to climatic conditions. The “adult” producing stage may last until the trees are 25 to 30 years old. All phenotypic measurements in the present experiment were carried out between 6 months and 7 years after planting, i.e., during the “young” stage and at the beginning of the “adult” stage.

For the first 6 years, only growth traits were periodically measured on all living trees of the field trial, namely the (i) height of trees taken at the insertion of the highest leaf on the trunk, and the (ii) circumference of the trunk measured 1 m above the soil level. In the seventh year after planting, all trees with a circumference equal or superior to 25 cm were tapped once every 3 days for six consecutive months. The cumulative production of coagulated latex was assessed three times during this tapping period.

**Fig. 4:**
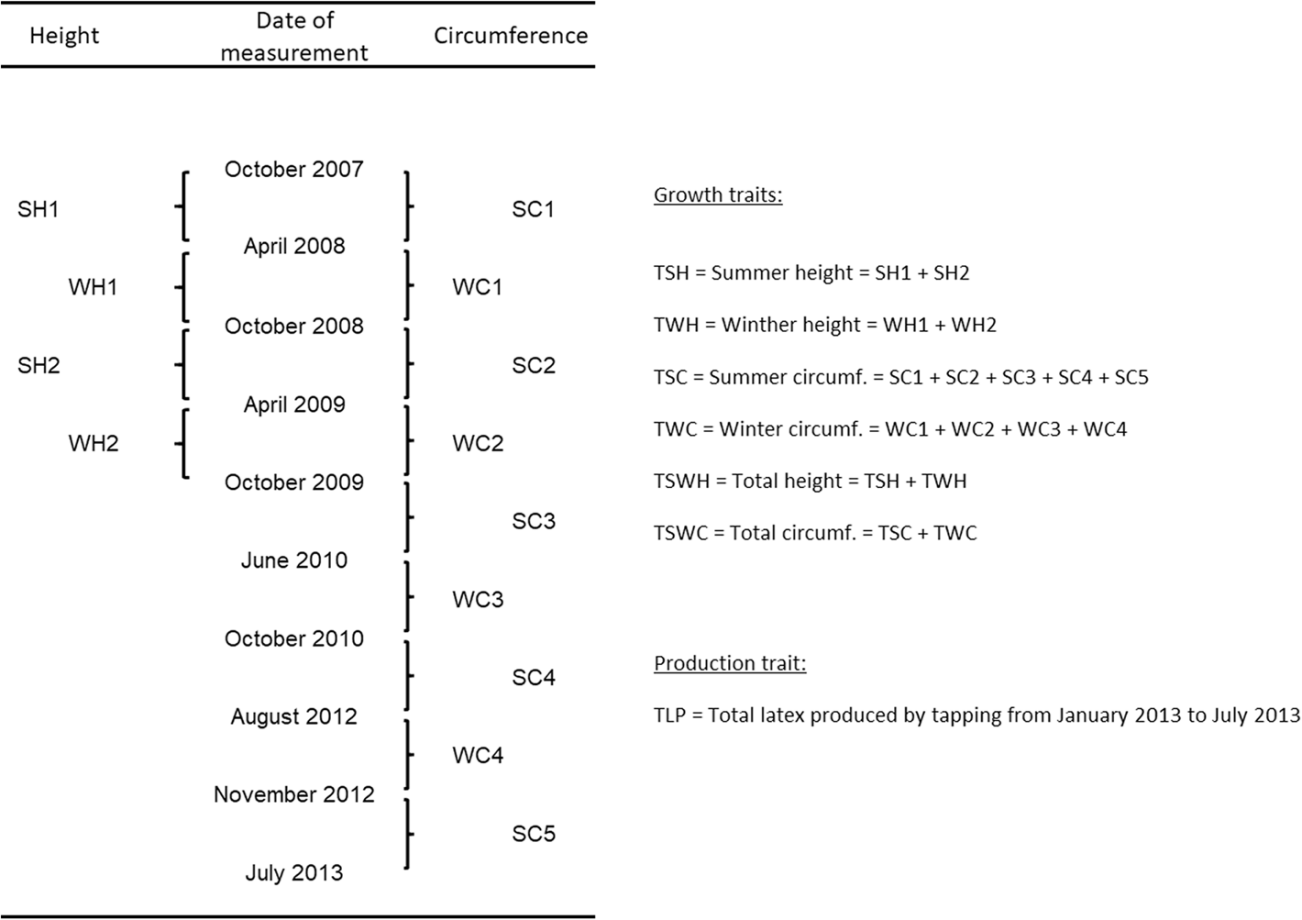
Dates of growth and production measurements and definition of phenotypic traits

Height and circumference increments were defined as the difference between measurements obtained in October of year N and April of year N + 1 (season summer – S) and April of year N and October of year N (season winter – W) as described by Souza et al. [14]. Overall, twenty traits were obtained and used for phenotypic analyses (see Fig. 4): height increments during summer (SH1, SH2 and TSH), winter (WH1, WH2 and TWH), and total height increment (TSWH); circumference increments during summer (SC1, SC2, SC3, SC4, SC5 and TSC), winter (WC1, WC2, WC3, WC4 and TWC) and total circumference increment (TSWC); and TLP. The six consecutive months of tapping for assessing latex production totally corresponded to the period during which SC5 was estimated; SC5 should be considered the circumference increment under tapping conditions.

### Phenotypic analyses

A linear mixed model approach was used to analyze all phenotypic traits. The best linear unbiased prediction (BLUP) of each F1 individual was used to perform QTL mapping. To reduce the complexity of the model adjustment, height and circumference analyses were performed separately for each session, summer or winter (model 1). Latex production analyses were performed as repeated measures over time with three evaluations of cumulative or total latex production (model 2):

1 $$ {y}_{ijklw}=\mu +{b}_i+{l}_j+{c}_k+{p}_w+{g}_l+{\varepsilon}_{ijklw} $$

2 $$ {y}_{ijklm}=\mu +{b}_i+{l}_j+{c}_k+{a}_m+{g}_l+{\varepsilon}_{ijklm} $$

where *y*_*ijklw*_ is the phenotypic observation (height or circumference) related to block *i*, line *j*, column *k*, genotype *l* and planting date *w*; *y*_*ijklm*_ is the phenotypic observation (latex production) related to block *i*, line *j*, column *k*, genotype *l* and circumference *m*; *μ* is the average; *b*_*i*_ is the effect of block *i*; *l*_*j*_ is the effect of line *j*; *c*_*k*_ is the effect of column *k*; *p*_*w*_ is the effect of planting date *w*; *a*_*m*_ is the effect of co-variable circumference *m*; *g*_*l*_ is the effect of genotype *l*; and *ε*_*ijklw*_ and *ε*_*ijklm*_ are both residual terms. Except *g*_*l*_, all effects and residuals were considered fixed.

For growth traits (height and circumference), different variance-covariance structures were investigated for the genetic effects matrix accounting for interactions of individuals (clones) along the experimental blocks (***G*** = ***G***_*B*_ ⊗ ***I***). For latex production, different structures were tested for the genetic effects matrix accounting for repeated measures over time (***G*** = ***G***_*E*_ ⊗ ***I***). These structures were identity (ID), diagonal (DIAG)*,* heterogeneous compound symmetry (CS_Het_), factor analytic (FA), and unstructured (UNST), which are detailed by Pastina [45]. Given the best model for the genetic effects matrix, the structures ID, DIAG, auto-regressive of order 1 (AR), and heterogeneous auto-regressive of order 1 (AR_Het_) were investigated for residual effects matrices related to lines and columns (***R***_*L*_ ⊗ ***R***_*C*_) of the experiment. The use of lines and columns as co-variables in models (1) and (2) as well as the incorporation of their tested residual matrices provided additional variation control. Components of genetic and residual variances and covariances for the described structures were estimated using the restricted maximum likelihood method (REML) [46]. Models were compared via AIC [47], and all analyses were performed using the R version of *ASReml* software [48].

### SSR marker genotyping

SSR markers were obtained mainly from enriched genomic libraries or expressed sequence tag (EST) libraries (Table 1). A few markers came from suppression subtractive hybridization (SSH) cDNA libraries [49] or were directly identified in the GenBank Nucleotide public database [50, 51]. Amplifications by polymerase chain reaction (PCR) were performed as described in Table 1. Most of the SSR amplicons generated by PCR were separated by migration on denaturing acrylamide gels and visualized by two alternative techniques, silver staining [52] or fluorescence, using a LICOR 4300 DNA analyzer as previously described [53]. A few SSR markers were characterized with an Advance FS96 dsDNA Fragment Analysis instrument (Advanced Analytical Technologies Inc., USA) or by capillary electrophoresis on an ABI 3500xL (Applied Biosystem) instrument [54].

### SNP marker development and genotyping

SNPs developed and presented in previous rubber tree studies [16, 17, 22] were evaluated for polymorphisms in the parental clones PR 255 and PB 217 and selected as molecular markers. A subset of putative SNPs identified from de novo transcriptome assemblies [16, 22] and EST full-length libraries [17] for rubber tree was used to develop novel molecular markers (Additional file 2: Table S6). Two different genotyping technologies were used: (1) the Sequenom MassARRAY® platform (AgenaBio, San Diego, CA), based on mass spectrometry genotyping, and (2) the Fluidigm® platform (South San Francisco, CA), based on KASP™ chemistry.

#### Sequenom MassARRAY® platform

A total of 243 targeted SNPs from 226 sequences (Additional file 3: Table S7), 29 previously validated SNPs [16, 17] and 214 SNPs predicted from de novo transcriptome assembly [16] and full-length ESTs [17], were submitted to the MassARRAY Assay Design program (Agena Bioscience®, San Diego, CA). This program designs capture primers (PCR reaction), which amplify the targeted region, and the extension primer, which binds immediately adjacent to the SNP. Primer design was performed using the following parameters: (1) high plex preset with a multiplex level = 24; (2) amplicon length varying from 80 to 200 bp, and (3) number of iterations = 10. Genotyping reactions were performed following the manufacturer’s instructions with DNA input concentrations varying from 1 to 3 ng/μl. These SNPs are listed in Additional file 2.

#### KASP™ chemistry technology

This technology is based on PCR with competitive allele-specific forward primers and enables the visualization of SNP sites in either homozygous or heterozygous states. Polymorphic SNPs on parental clones of the F1 population were screened on a LightCycler® 480 instrument with KASP™ chemistry. Genotyping of 192 progeny individuals of the F1 population was performed on a Fluidigm® instrument with the same chemistry with 96 SNPs using a nanofluidic 96.96 Dynamic Array IFC instrument (Integrated Fluidic Circuit, Fluidigm Corp.).

### Integrated genetic linkage map

A total of 251 individuals (of 270 initially obtained) of the PR 255 x PB 217 population and 555 molecular markers, 364 SSRs and 191 SNPs (Table 1), were used for construction of the integrated genetic linkage map. Marker segregation was assessed using a Chi-square test and Bonferroni correction for multiple tests. The integrated genetic linkage map was constructed using a multipoint approach based on hidden Markov models (HMM) [18], which are implemented in the freely available *OneMap* package [55] within R statistical software [56]. Initially, recombination fractions and linkage phases were simultaneously estimated for each pair of markers, and linkage groups (LGs) were obtained based on a maximum recombination fraction of 0.35 and a minimum LOD score of 4.50. A multipoint approach was used to order markers and re-estimate the recombination fractions into each LG [57, 58] using *OneMap*’s graphics to check for inconsistencies. Genetic distances were obtained using the Kosambi mapping function [59]. Final visualization of the map was obtained using *MapChart* software [60].

### QTL mapping

An extension of composite interval mapping (CIM) [61] for an outcrossing population was performed [19] using the integrated (multipoint) genetic linkage map and the predicted genotypic values of the traits obtained via *ASReml*. Initially, the genetic linkage map was scanned, and QTL searches were performed every 1 cM. In each position, the QTL model was fitted using the effects of molecular markers outside the mapping interval as cofactors, selected via stepwise regression. A window size of 15 cM was used, and 1000 permutations were performed based on two different approaches to declare significance [20, 21]. All genomic positions with LOD scores superior to the threshold with a significance level of 0.05 suggested the presence of a QTL. For these positions, three effects were marginally tested following a previously suggested approach [19]: (i) additive for the parent PR 255 (*α*_*p*_), (ii) additive for the parent PB 217 (*α*_*q*_), and (iii) dominance involving additive alleles from both parents (*δ*_*pq*_). The segregation pattern of the QTL and its linkage phase were estimated following the procedure developed by Gazaffi et al. [19].

## Additional files

Additional file 1: **Table S1** Summarized description of the genetic linkage map of the PR 255 x PB 217 F1 population. **Table S2.** Different variance-covariance structures for the genetic matrix related to height, circumference, and latex production traits. **Table S3.** Different variance-covariance structures for the residual matrix related to height, circumference, and latex production traits. **Table S4.** Selected models for the genetic and residual matrices related to height, circumference, and latex production traits. **Table S5.** Characteristics of the 111 QTLs mapped for all phenotypic traits. (DOCX 90 kb)

Additional file 2: **Table S6**. Characteristics of sHbCIR and sHbUNI SNP markers. (XLSX 20 kb)

Additional file 3: **Table S7.** Characteristics of Hb_seq SNP markers and primers defined for genotyping with the Sequenom MassARRAY® platform. (XLSX 68 kb)

## Abbreviations

AIC: Akaike information criteria
AR: Auto-regressive of order 1
AR1_Het_: Heterogeneous auto-regressive of order 1
BLUP: Best linear unbiased prediction
CIM: Composite interval mapping
cM: CentiMorgan
CS_Het_: Heterogeneous compound symmetry
DIAG: Diagonal
EST: Expressed sequence tag
FA: Factor analytic
ID: Identity
LG: Linkage group
LOD: Logarithm of odds
MAS: Marker-assisted selection
PCR: Polymerase chain reaction
QTL: Quantitative trait loci
REML: Restricted maximum likelihood
SALB: South American leaf blight
SC: Circumference increment during summer
SH: Height increment during summer
SNP: Single-nucleotide polymorphism
SSH: Suppressive subtractive hybridization
SSR: Simple sequence repeat
TLP: Total latex production
TSC: Total circumference increment during summer
TSH: Total height increment during summer
TSWC: Total circumference increment
TSWH: Total height increment
TWC: Total circumference increment during winter
TWH: Total height increment during winter
UNST: Unstructured
WC: Circumference increment during winter
WH: Height increment during winter
