# Supplementary material for: QTL detection for growth and latex production in a full-sib rubber tree population cultivated under suboptimal climate conditions
Source: BMC Plant Biol. 2018 Oct 10;18:223. doi: 10.1186/s12870-018-1450-y (PMC6180592; doi:10.1186/s12870-018-1450-y)
Supplement: Supplementary file 1 — Table S1 Summarized description of the genetic linkage map of the PR 255 x PB 217 F1 population. Table S2. Different variance-covariance structures for the genetic matrix related to height, circumference, and latex production traits. Table S3. Different variance-covariance structures for the residual matrix related to height, circumference, and latex production traits. Table S4. Selected models for the genetic and residual matrices related to height, circumference, and latex production traits. Table S5. Characteristics of the 111 QTLs mapped for all phenotypic traits. (DOCX 90 kb) [file 12870_2018_1450_MOESM1_ESM.docx]

**Table S1. Summarized description of the genetic linkage map of the PR 255 x PB 217 F1 population.**

| Linkage Group  (LG) | Length  (cM) | Number of SSR markers | Number of SNP markers | Total number of markers | Average distance between markers (cM) |
| --- | --- | --- | --- | --- | --- |
| 1 | 182.4 | 25 | 11 | 36 | 5.21 |
| 2 | 228.5 | 21 | 9 | 30 | 7.88 |
| 3 | 206.7 | 22 | 5 | 27 | 7.95 |
| 4 | 126.9 | 11 | 8 | 19 | 7.05 |
| 5 | 220.5 | 22 | 10 | 32 | 7.11 |
| 6 | 180.5 | 18 | 13 | 31 | 6.02 |
| 7 | 182.8 | 20 | 9 | 29 | 6.53 |
| 8 | 235.0 | 25 | 4 | 29 | 8.39 |
| 9 | 155.7 | 15 | 7 | 22 | 7.41 |
| 10 | 302.6 | 35 | 12 | 47 | 6.58 |
| 11 | 200.6 | 10 | 10 | 20 | 10.56 |
| 12A | 181.5 | 13 | 6 | 19 | 10.08 |
| 12B | 19.1 | 0 | 3 | 3 | 9.55 |
| 13 | 188.2 | 18 | 5 | 23 | 8.55 |
| 14 | 285.4 | 28 | 14 | 42 | 6.96 |
| 15 | 168.5 | 18 | 5 | 23 | 7.66 |
| 16 | 166.8 | 18 | 3 | 21 | 8.34 |
| 17 | 115.4 | 13 | 5 | 18 | 6.79 |
| 18A | 159.3 | 16 | 5 | 21 | 7.97 |
| 18B | 10.8 | 6 | 0 | 6 | 2.17 |
| 19 | 3.1 | 0 | 2 | 2 | 3.10 |
| 20 | 3.1 | 0 | 3 | 3 | 3.10 |
| 21 | 1.8 | 0 | 2 | 2 | 1.80 |
|  |  |  |  |  |  |
| **Total** | **3,525.2** | **354** | **151** | **505** | **7.44** |

**Table S2. Different variance-covariance structures for the genetic matrix related to height, circumference, and latex production traits.**

| Trait | Genetic matrix | Tested model | AIC | | | | | | |
| --- | --- | --- | --- | --- | --- | --- | --- | --- | --- |
|  |  |  | SH1 | SH2 | TSH | WH1 | WH2 | TWH | TSWH |
| Height | $\boldsymbol{G}_{B}\otimes\boldsymbol{I}$ | ID $\otimes$ ID | **27,832.90** | 29,905.16 | **33,249.39** | 24,302.48 | 26,748.81 | 29,108.17 | **33,641.59** |
|  |  | DIAG $\otimes$ ID | 27,834.22 | 29,905.77 | 33,255.29 | 24,300.81 | **26,742.08** | 29,104.38 | 33,645.26 |
|  |  | CS_Het_ $\otimes$ ID | NC | NC | NC | NC | NC | NC | NC |
|  |  | FA $\otimes$ ID | 27,838.93 | **29,899.30** | 33,261.55 | **24,295.00** | 26,748.08 | **29,100.80** | 33,644.82 |
|  |  | UNST $\otimes$ ID | NC | NC | NC | NC | NC | NC | NC |
|  |  |  |  |  |  |  |  |  |  |
|  |  |  | SC1 | SC2 | SC3 | SC4 | SC5 | TSC |  |
| Circumfer. | $\boldsymbol{G}_{B}\otimes\boldsymbol{I}$ | ID $\otimes$ ID | 19,380.01 | **23,166.45** | **24,445.07** | 27,782.71 | 20,735.00 | **30,688.38** |  |
|  |  | DIAG $\otimes$ ID | **19,376.46** | 23,170.78 | 24,450.87 | **27,772.84** | 20,733.06 | 30,693.84 |  |
|  |  | CS_Het_ $\otimes$ ID | NC | NC | NC | NC | NC | NC |  |
|  |  | FA $\otimes$ ID | 19,377.81 | NC | 24,454.63 | NC | **20,731.35** | NC |  |
|  |  | UNST $\otimes$ ID | NC | NC | NC | NC | NC | NC |  |
|  |  |  |  |  |  |  |  |  |  |
|  |  |  | WC1 | WC2 | WC3 | WC4 | TWC | TSWC |  |
| Circumfer. | $\boldsymbol{G}_{B}\otimes\boldsymbol{I}$ | ID $\otimes$ ID | **13,103.83** | 18,813.49 | 14,676.96 | 15,478.54 | **22,310.79** | **31,350.97** |  |
|  |  | DIAG $\otimes$ ID | 13,109.42 | **18,807.09** | **14,649.24** | 15,477.64 | 22,311.81 | 31,355.49 |  |
|  |  | CS_Het_ $\otimes$ ID | NC | NC | NC | NC | NC | NC |  |
|  |  | FA $\otimes$ ID | NC | 18,808.06 | 14,653.52 | **15,475.84** | NC | NC |  |
|  |  | UNST $\otimes$ ID | NC | NC | NC | NC | NC | NC |  |
|  |  |  |  |  |  |  |  |  |  |
|  |  |  | TLP |  |  |  |  |  |  |
| Latex Prod. | $\boldsymbol{G}_{E}\otimes\boldsymbol{I}$ | DIAG $\otimes$ ID | 31,317.74 |  |  |  |  |  |  |
|  |  | CS_Het_ $\otimes$ ID | 31,318.36 |  |  |  |  |  |  |
|  |  | AR1_Het_ $\otimes$ ID | **31,314.53** |  |  |  |  |  |  |
|  |  | FA $\otimes$ ID | NC |  |  |  |  |  |  |
|  |  | UNST $\otimes$ ID | 31,321.39 |  |  |  |  |  |  |

**Table S3. Different variance-covariance structures for the residual matrix related to height, circumference, and latex production traits.**

| Trait | Genetic matrix | Tested model | AIC^1^ | | | | | | |
| --- | --- | --- | --- | --- | --- | --- | --- | --- | --- |
|  |  |  | SH1 | SH2 | TSH | WH1 | WH2 | TWH | TSWH |
| Height | $\boldsymbol{R}_{L}\otimes\boldsymbol{R}_{C}$ | DIAG $\otimes$ ID | **27,688.11** | 29,952.79 | 33,009.46 | 24,348.41 | 26,795.09 | **29,062.68** | **33,465.93** |
|  |  | DIAG $\otimes$ DIAG | NC | NC | NC | NC | NC | NC | NC |
|  |  | ID $\otimes$ DIAG | 27,840.46 | 29,941.95 | 33,091.15 | 24,302.28 | 26,808.75 | 29,138.13 | 33,562.72 |
|  |  | AR1 $\otimes$ ID | 27,833.83 | 29,900.67 | 33,251.35 | **24,290.68** | **26,744.08** | 29,099.31 | 33,643.55 |
|  |  | AR1_Het_ $\otimes$ ID | 27,688.20 | 29,954.22 | 33,011.14 | 24,340.44 | 26,797.09 | 29,063.49 | 33,467.91 |
|  |  | DIAG $\otimes$ AR1 | 27,689.62 | 29,952.84 | **33,007.47** | 24,350.25 | 26,796.59 | 29,064.56 | 33,465.99 |
|  |  | DIAG $\otimes$ AR1_Het_ | NC | NC | NC | NC | NC | NC | NC |
|  |  | AR1 $\otimes$ AR1 | 27,834.67 | **29,900.20** | 33,251.18 | 24,292.61 | 26,745.55 | 29,100.77 | 33,644.32 |
|  |  | AR1 $\otimes$ AR1_Het_ | 27,842.23 | 29,942.17 | 33,092.62 | 24,302.35 | 26,812.28 | 29,138.86 | 33,565.60 |
|  |  | AR1_Het_ $\otimes$ AR1 | 27,690.00 | 29,953.59 | 33,009.45 | 24,342.35 | 26,798.56 | 29,065.13 | 33,467.75 |
|  |  | AR1_Het_ $\otimes$ AR1_Het_ | NC | NC | NC | NC | NC | NC | NC |
|  |  |  |  |  |  |  |  |  |  |
|  |  |  | SC1 | SC2 | SC3 | SC4 | SC5 | TSC |  |
| Circumfer. | $\boldsymbol{R}_{L}\otimes\boldsymbol{R}_{C}$ | DIAG $\otimes$ ID | 19,245.46 | 23,200.12 | 24,535.06 | 27,710.71 | 20,768.06 | 30,757.96 |  |
|  |  | DIAG $\otimes$ DIAG | NC | NC | NC | NC | NC | NC |  |
|  |  | ID $\otimes$ DIAG | 19,289.66 | 23,201.78 | 24,476.16 | 27,743.68 | 20,737.99 | 30,744.88 |  |
|  |  | AR1 $\otimes$ ID | 19,375.51 | **23,168.15** | 24,443.31 | 27,764.11 | 20,724.93 | **30,685.71** |  |
|  |  | AR1_Het_ $\otimes$ ID | **19,244.76** | 23,202.03 | 24,533.55 | **27,705.92** | 20,764.22 | 30,755.80 |  |
|  |  | DIAG $\otimes$ AR1 | 19,247.09 | 23,200.40 | 24,532.04 | 27,712.66 | 20,761.13 | 30,759.19 |  |
|  |  | DIAG $\otimes$ AR1_Het_ | NC | NC | NC | NC | NC | NC |  |
|  |  | AR1 $\otimes$ AR1 | 19,376.47 | 23,168.61 | **24,441.25** | 27,766.10 | 20,718.53 | 30,687.02 |  |
|  |  | AR1 $\otimes$ AR1_Het_ | 19,289.79 | 23,204.47 | 24,472.71 | 27,735.85 | **20,718.50** | 30,744.83 |  |
|  |  | AR1_Het_ $\otimes$ AR1 | 19,246.03 | 23,202.40 | 24,532.25 | 27,707.73 | 20,752.78 | 30,755.91 |  |
|  |  | AR1_Het_ $\otimes$ AR1_Het_ | NC | NC | NC | NC | NC | NC |  |
|  |  |  |  |  |  |  |  |  |  |
|  |  |  |  |  |  |  |  |  |  |

**Table S3. Different variance-covariance structures for the residual matrix related to height, circumference, and latex production traits (continued).**

| Trait | Genetic matrix | Tested model | AIC^1^ | | | | | | |
| --- | --- | --- | --- | --- | --- | --- | --- | --- | --- |
|  |  |  | WC1 | WC2 | WC3 | WC4 | TWC | TSWC |  |
| Circumfer. | $\boldsymbol{R}_{L}\otimes\boldsymbol{R}_{C}$ | DIAG $\otimes$ ID | **13,082.74** | **18,791.80** | 14,095.60 | 15,473.86 | 22,339.97 | 31,405.34 |  |
|  |  | DIAG $\otimes$ DIAG | NC | NC | NC | NC | NC | NC |  |
|  |  | ID $\otimes$ DIAG | 13,176.17 | 18,835.40 | 14,615.21 | 15,481.02 | 22,346.58 | 31,410.52 |  |
|  |  | AR1 $\otimes$ ID | 13,104.94 | 18,807.47 | 14,639.45 | 15,473.99 | **22,303.26** | 31,346.40 |  |
|  |  | AR1_Het_ $\otimes$ ID | 13,083.99 | 18,793.35 | 14,096.11 | **15,472.29** | 22,333.32 | 31,400.67 |  |
|  |  | DIAG $\otimes$ AR1 | 13,084.18 | 18,793.13 | **14,083.79** | 15,475.75 | 22,341.92 | 31,405.44 |  |
|  |  | DIAG $\otimes$ AR1_Het_ | NC | NC | NC | NC | NC | NC |  |
|  |  | AR1 $\otimes$ AR1 | 13,106.88 | 18,809.44 | 14,594.16 | 15,475.08 | 22,305.25 | **31,346.33** |  |
|  |  | AR1 $\otimes$ AR1_Het_ | 13,179.00 | 18,838.57 | 14,562.36 | 15,481.43 | 22,340.99 | 31,407.39 |  |
|  |  | AR1_Het_ $\otimes$ AR1 | 13,085.62 | 18,794.86 | 14,085.58 | 15,473.70 | 22,335.15 | 31,398.60 |  |
|  |  | AR1_Het_ $\otimes$ AR1_Het_ | NC | NC | NC | NC | NC | NC |  |
|  |  |  |  |  |  |  |  |  |  |
|  |  |  | TLP |  |  |  |  |  |  |
| Latex Prod. | $\boldsymbol{R}_{L}\otimes\boldsymbol{R}_{C}$ | DIAG $\otimes$ ID | 31,343.36 |  |  |  |  |  |  |
|  |  | ID $\otimes$ DIAG | 31,306.16 |  |  |  |  |  |  |
|  |  | DIAG $\otimes$ DIAG | NC |  |  |  |  |  |  |
|  |  | AR1 $\otimes$ ID | 31,298.66 |  |  |  |  |  |  |
|  |  | AR1_Het_ $\otimes$ ID | 31,328.17 |  |  |  |  |  |  |
|  |  | DIAG $\otimes$ AR1 | 31,341.71 |  |  |  |  |  |  |
|  |  | DIAG $\otimes$ AR1_Het_ | NC |  |  |  |  |  |  |
|  |  | AR1 $\otimes$ AR1 | 31,297.25 |  |  |  |  |  |  |
|  |  | AR1 $\otimes$ AR1_Het_ | **31,288.75** |  |  |  |  |  |  |
|  |  | AR1_Het_ $\otimes$ AR1 | 31,329.16 |  |  |  |  |  |  |
|  |  | AR1_Het_ $\otimes$ AR1_Het_ | NC |  |  |  |  |  |  |

^1^AIC values correspond to the mixed models with the most likely genetic matrix and the tested residual matrix.

**Table S4. Selected models for the genetic and residual matrices related to height, circumference, and latex production traits.**

| Trait | Genetic matrix | Selected model | Residual matrix | Selected model | Number of parameters | AIC |
| --- | --- | --- | --- | --- | --- | --- |
| SH1 | $\boldsymbol{G}_{B}\otimes\boldsymbol{I}$ | ID $\otimes$ ID | $\boldsymbol{R}_{L}\otimes\boldsymbol{R}_{C}$ | DIAG $\otimes$ ID | 83 | 27,688.11 |
| SH2 |  | FA $\otimes$ ID |  | ID $\otimes$ ID | 10 | 29,899.30 |
| TSH |  | ID $\otimes$ ID |  | DIAG $\otimes$ AR1 | 84 | 33,007.47 |
| WH1 |  | FA $\otimes$ ID |  | AR1 $\otimes$ ID | 11 | 24,290.68 |
| WH2 |  | DIAG $\otimes$ ID |  | ID $\otimes$ ID | 6 | 26,742.08 |
| TWH |  | FA $\otimes$ ID |  | DIAG $\otimes$ ID | 90 | 29,062.68 |
| TSWH |  | ID $\otimes$ ID |  | DIAG $\otimes$ ID | 83 | 33,465.93 |
|  |  |  |  |  |  |  |
| SC1 | $\boldsymbol{G}_{B}\otimes\boldsymbol{I}$ | DIAG $\otimes$ ID | $\boldsymbol{R}_{L}\otimes\boldsymbol{R}_{C}$ | AR1_Het_ $\otimes$ ID | 87 | 19,244.76 |
| SC2 |  | ID $\otimes$ ID |  | ID $\otimes$ ID | 3 | 23,166,45 |
| SC3 |  | ID $\otimes$ ID |  | AR1 $\otimes$ AR1 | 5 | 24,441.25 |
| SC4 |  | DIAG $\otimes$ ID |  | AR1_Het_ $\otimes$ ID | 87 | 27,705.92 |
| SC5 |  | FA $\otimes$ ID |  | AR1 $\otimes$ AR1_Het_ | 68 | 20,718.50 |
| TSC |  | ID $\otimes$ ID |  | AR1 $\otimes$ ID | 4 | 30,685.71 |
| WC1 |  | ID $\otimes$ ID |  | DIAG $\otimes$ ID | 83 | 13,082.74 |
| WC2 |  | DIAG $\otimes$ ID |  | DIAG $\otimes$ ID | 86 | 18,791.80 |
| WC3 |  | DIAG $\otimes$ ID |  | DIAG $\otimes$ AR1 | 87 | 14,083.79 |
| WC4 |  | FA $\otimes$ ID |  | AR1_Het_ $\otimes$ ID | 91 | 15,472.29 |
| TWC |  | ID $\otimes$ ID |  | AR1 $\otimes$ ID | 4 | 22,303.26 |
| TSWC |  | ID $\otimes$ ID |  | AR1 $\otimes$ AR1 | 5 | 31,346.33 |
|  |  |  |  |  |  |  |
| TLP | $\boldsymbol{G}_{E}\otimes\boldsymbol{I}$ | AR1_Het_ $\otimes$ ID |  | AR1 $\otimes$ AR1_Het_ | 98 | 31,288.75 |

These models were selected according to the lowest value of the AIC, which were calculated using both genetic and residual matrices in the statistical model.

**Table S5.** Characteristics of the 111 QTLs mapped for all phenotypic traits: *R²*, LOD score, additive and dominance effects, segregation type.

| QTL | Trait | Flanking Markers | Linkage Group (LG) | Position  in cM | Global LOD | *R^2^* | α_PR 255_  (LOD) | α_PB 217_  (LOD) | δ_PR 255 x PB 217_  (LOD) | Segregation |
| --- | --- | --- | --- | --- | --- | --- | --- | --- | --- | --- |
| 1 | TWH | sHbUNI0515_S | 1 | 0.00 | 5.365 | 2.363 | 3.149  (4.363) | 2.193  (2.358) | 3.055  (4.162) | 3:1 |
|  | TWC | sHbUNI0515_S | 1 | 0.00 | 4.016 | 1.161 | 1.246  (1.889) | 1.753  (3.202) | 1.841  (3.604) | 3:1 |
|  | SC1 | sHbUNI0515_S – g01A2746 | 1 | 2.00 | 3.629 | 0.932 | -1.169  (2.538) | -0.993  (0.829) | 0.188  (0.151) | 1:1 |
| 2 | WC1 | Hb_seq_06_2 | 1 | 30.32 | 14.344 | 1.799 | -0.642  (7.286) | -0.800  (8.310) | 0.509  (8.769) | 1:1:1:1 |
| 3 | SC3 | HBE110 | 1 | 49.73 | 4.463 | 3.394 | -0.658  (0.712) | -0.792  (1.183) | 1.202  (2.471) | 1:2:1 |
| 4 | TLP | HBE225 | 1 | 59.00 | 7.735 | 4.219 | 10.956  (1.331) | 22.548  (6.015) | -11.302  (1.446) | 1:1:1:1 |
| 5 | SC4 | A2419 | 2 | 3.96 | 10.870 | 13.699 | -1.847  (4.793) | 0.139  (0.029) | -2.077  (5.918) | 1:2:1 |
| 6 | WH1 | A2348 – HB_seq_38_2 | 2 | 18.00 | 10.890 | 1.805 | 5.318  (8.483) | 5.865  (9.658) | 6.309  (10.581) | 3:1 |

**Table S5.** Characteristics of the 111 QTLs mapped for all phenotypic traits: *R²*, LOD score, additive and dominance effects, segregation type.

| QTL | Trait | Flanking Markers | Linkage Group (LG) | Position  in cM | Global LOD | *R^2^* | α_PR 255_  (LOD) | α_PB 217_  (LOD) | δ_PR 255 x PB 217_  (LOD) | Segregation |
| --- | --- | --- | --- | --- | --- | --- | --- | --- | --- | --- |
| 6 | WC1 | A2348 – HB_seq_38_2 | 2 | 18.00 | 18.689 | 1.972 | 1.106  (15.647) | 1.187  (17.313) | 1.249  (18.190) | 3:1 |
| 7 | SC5 | sHbCIR0100 | 2 | 32.70 | 4.623 | 6.877 | -0.929  (3.038) | -0.757  (2.564) | -0.314  (0.371) | 1:2:1 |
|  | WC4 | sHbCIR0100 | 2 | 32.70 | 8.636 | 7.221 | -0.454  (4.998) | -0.388  (5.179) | -0.242  (1.723) | 3:1 |
| 8 | SC3 | A2419 | 2 | 39.59 | 11.116 | 0.472 | -2.102  (7.578) | 0.236  (0.108) | -1.604  (4.336) | 1:2:1 |
|  | TSC | A2419 | 2 | 39.59 | 17.732 | 14.426 | -7.189  (11.365) | 0.174  (0.007) | -6.086  (7.687) | 1:2:1 |
|  | SC3 | A2419 – sHbUNI0513 | 2 | 40.00 | 11.081 | 0.537 | -2.087  (7.486) | 0.244  (0.114) | -1.619  (4.368) | 1:2:1 |
|  | SC2 | A2419 – sHbUNI0513 | 2 | 43.00 | 5.125 | 5.567 | -0.712  (1.802) | 0.251  (0.228) | -0.989  (3.075) | 1:2:1 |
| 9 | WC3 | g02T2607 | 2 | 84.58 | 5.541 | 4.515 | 0.227  (4.490) | 0.125  (1.409) | -0.017  (0.026) | 1:2:1 |

**Table S5.** Characteristics of the 111 QTLs mapped for all phenotypic traits: *R²*, LOD score, additive and dominance effects, segregation type.

| QTL | Trait | Flanking Markers | Linkage Group (LG) | Position  in cM | Global LOD | *R^2^* | α_PR 255_  (LOD) | α_PB 217_  (LOD) | δ_PR 255 x PB 217_  (LOD) | Segregation |
| --- | --- | --- | --- | --- | --- | --- | --- | --- | --- | --- |
| 9 | TWH | g02T2607 – Hb_seq_138 | 2 | 88.00 | 5.369 | 5.539 | -2.313  (4.185) | 1.001  (0.699) | -0.578  (0.224) | 1:1 |
| 10 | TLP | g02t182 – SSH197 | 2 | 159.00 | 7.637 | 4.391 | 21.299  (4.084) | 19.744  (3.535) | -12.484  (1.321) | 3:1 |
| 11 | WC2 | EHBc92 | 2 | 205.38 | 4.742 | 2.565 | 0.057  (0.026) | 0.304  (0.496) | 0.801  (4.412) | 1:1 |
| 12 | SC4 | g10a262 – HBE134 | 3 | 93.00 | 7.034 | 2.394 | 2.263  (3.516) | 1.576  (4.046) | -1.121  (1.084) | 3:1 |
| 13 | TLP | EHBp23a | 3 | 101.13 | 5.268 | 1.416 | 14.598  (2.398) | -12.227  (1.673) | 10.678  (1.272) | 3:1 |
|  | WC2 | HB180 | 3 | 105.44 | 5.592 | 3.473 | 0.566  (2.923) | -0.361  (1.245) | -0.544  (2.722) | 3:1 |
| 14 | WC1 | HB24 – sHbUNI0502 | 3 | 116.00 | 5.900 | 4.451 | 0.382  (4.927) | -0.102  (0.367) | -0.284  (2.264) | 1:2:1 |
|  | TWC | HB24 – sHbUNI0502 | 3 | 117.00 | 5.445 | 4.828 | 1.508  (4.499) | -0.434  (0.285) | -1.120  (2.087) | 1:2:1 |

**Table S5.** Characteristics of the 111 QTLs mapped for all phenotypic traits: *R²*, LOD score, additive and dominance effects, segregation type.

| QTL | Trait | Flanking Markers | Linkage Group (LG) | Position  in cM | Global LOD | *R^2^* | α_PR 255_  (LOD) | α_PB 217_  (LOD) | δ_PR 255 x PB 217_  (LOD) | Segregation |
| --- | --- | --- | --- | --- | --- | --- | --- | --- | --- | --- |
| 15 | SH2 | HB176 – EHBc57 | 3 | 164.00 | 3.665 | 4.169 | -1.028  (0.708) | -0.174  (0.023) | 2.200  (3.092) | 1:1 |
| 16 | WC4 | EHBc57 | 3 | 170.95 | 5.930 | 6.413 | 0.260  (2.399) | -0.032  (0.038) | 0.320  (3.662) | 1:2:1 |
| 17 | SC3 | HBE121 – A2749 | 3 | 184.00 | 8.542 | 0.255 | -0.573  (0.566) | 0.711  (0.889) | 2.066  (6.848) | 1:1:1:1 |
|  | SC3 | HBE121 – A2749 | 3 | 185.00 | 8.502 | 0.210 | -0.576  (0.558) | 0.699  (0.857) | 2.093  (6.814) | 1:1:1:1 |
|  | TSC | HBE121 – A2749 | 3 | 186.00 | 4.940 | 3.555 | -1.503  (0.468) | -0.524  (0.060) | 5.010  (4.571) | 1:1 |
| 18 | WH2 | g04A2402 – sHbCIR0057 | 4 | 86.00 | 3.485 | 2.826 | 0.549  (1.105) | 0.441  (0.770) | 0.746  (1.771) | 1:2:1 |
| 19 | TLP | sHbCIR0057 | 4 | 97.15 | 13.252 | 8.678 | 14.391  (2.077) | -32.543  (12.118) | 8.657  (0.772) | 1:1:1:1 |
| 20 | TWH | g04a385 | 4 | 109.97 | 4.056 | 3.425 | 1.765  (2.502) | 1.467  (1.739) | 0.643  (0.337) | 1:2:1 |

**Table S5.** Characteristics of the 111 QTLs mapped for all phenotypic traits: *R²*, LOD score, additive and dominance effects, segregation type.

| QTL | Trait | Flanking Markers | Linkage Group (LG) | Position  in cM | Global LOD | *R^2^* | α_PR 255_  (LOD) | α_PB 217_  (LOD) | δ_PR 255 x PB 217_  (LOD) | Segregation |
| --- | --- | --- | --- | --- | --- | --- | --- | --- | --- | --- |
| 21 | WC3 | sHbCIR0066 | 5 | 17.42 | 3.449 | 2.730 | -0.086  (0.506) | 0.182  (2.776) | 0.043  (0.121) | 1:1 |
| 22 | WC4 | g05a390 | 5 | 86.99 | 9.921 | 8.525 | 0.497  (8.207) | 0.248  (2.537) | 0.012  (0.004) | 1:1:1:1 |
| 23 | TWC | HB190 | 5 | 93.59 | 5.554 | 5.128 | 1.409  (3.958) | 0.690  (0.979) | 0.795  (1.289) | 3:1 |
| 24 | WH1 | g05a491 | 5 | 148.29 | 5.423 | 1.728 | 0.992  (1.277) | 2.106  (4.156) | 0.382  (0.203) | 1:2:1 |
| 25 | SH2 | EHBmu5 | 5 | 161.15 | 5.243 | 3.235 | -1.619  (1.391) | 2.162  (4.722) | -1.842  (3.251) | 1:2:1 |
| 26 | TSH | g05A2723 | 5 | 212.22 | 6.604 | 5.143 | 2.760  (4.050) | 2.403  (3.118) | 1.067  (0.645) | 1:2:1 |
| 27 | SH1 | HB_seq_12 | 5 | 218.51 | 5.543 | 3.954 | 0.802  (1.099) | 1.356  (3.262) | 0.952  (1.488) | 3:1 |
| 28 | WH1 | EHBc48 | 6 | 33.78 | 6.748 | 1.386 | 0.401  (0.231) | -7.523  (6.104) | -0.826  (0.197) | 1:1 |

**Table S5.** Characteristics of the 111 QTLs mapped for all phenotypic traits: *R²*, LOD score, additive and dominance effects, segregation type.

| QTL | Trait | Flanking Markers | Linkage Group (LG) | Position  in cM | Global LOD | *R^2^* | α_PR 255_  (LOD) | α_PB 217_  (LOD) | δ_PR 255 x PB 217_  (LOD) | Segregation |
| --- | --- | --- | --- | --- | --- | --- | --- | --- | --- | --- |
| 29 | SC5 | HBE122 – HBE53 | 6 | 50.00 | 5.646 | 3.477 | -1.081  (1.502) | -1.606  (3.781) | -0.291  (0.402) | 1:2:1 |
| 30 | SH1 | Hb_seq_19_1 – g06T2449 | 6 | 131.00 | 6.389 | 6.751 | -1.569  (3.288) | -1.485 (3.232) | -0.160  (0.039) | 1:2:1 |
| 31 | TLP | g06T2449 – Hb_seq_128 | 6 | 139.00 | 6.717 | 1.172 | -5.454  (0.324) | 15.565  (2.427) | 25.484  (5.370) | 1:2:1 |
|  | SH2 | Hb_seq_128 | 6 | 142.76 | 4.915 | 3.453 | 1.992  (3.564) | -0.913  (0.657) | 0.823  (0.605) | 1:1 |
| 32 | WH2 | EHBp5 – sHbCIR0067 | 6 | 166.00 | 6.460 | 5.244 | 0.283  (0.338) | -1.004  (3.657) | -1.079  (3.826) | 1:2:1 |
| 33 | TSC | sHbCIR0262 – HBE49 | 6 | 174.00 | 4.161 | 2.895 | 4.085  (3.139) | -2.092  (0.640) | -0.987  (0.126) | 1:1 |
| 34 | SC2 | HBE49 | 6 | 180.49 | 6.021 | 4.900 | 1.236  (5.568) | -0.451  (0.344) | -0.195  (0.068) | 1:1 |
| 35 | WC2 | HBE116 | 7 | 9.59 | 3.731 | 2.731 | -0.141  (0.163) | 0.497  (2.117) | -0.434  (1.503) | 1:2:1 |

**Table S5.** Characteristics of the 111 QTLs mapped for all phenotypic traits: *R²*, LOD score, additive and dominance effects, segregation type.

| QTL | Trait | Flanking Markers | Linkage Group (LG) | Position  in cM | Global LOD | *R^2^* | α_PR 255_  (LOD) | α_PB 217_  (LOD) | δ_PR 255 x PB 217_  (LOD) | Segregation |
| --- | --- | --- | --- | --- | --- | --- | --- | --- | --- | --- |
| 36 | SC2 | g07A2429 | 7 | 121.73 | 4.233 | 3.207 | 0.532  (1.017) | -0.461  (0.788) | 0.834  (2.652) | 1:2:1 |
| 37 | SH2 | Hb_seq_161 | 8 | 10.04 | 5.758 | 3.279 | 1.373  (1.494) | -1.636  (2.807) | -1.671  (3.059) | 3:1 |
| 38 | TWH | g08A2442 | 8 | 24.92 | 4.852 | 3.849 | 2.056  (0.939) | 2.125  (3.248) | 0.032  (0.000) | 1:2:1 |
| 39 | WC4 | Hb_seq_171 | 8 | 57.95 | 4.150 | 4.620 | -0.128  (0.491) | 0.118  (0.452) | 0.291  (3.016) | 1:1 |
|  | WC3 | Hb_seq_171 – g08A66 | 8 | 60.00 | 4.936 | 4.705 | -0.221  (3.709) | -0.126  (1.285) | -0.023  (0.037) | 1:2:1 |
|  | TWC | Hb_seq_171 – g08A66 | 8 | 60.00 | 5.505 | 3.654 | -1.259  (3.063) | -0.730  (1.081) | 1.269  (2.883) | 3:1 |
| 40 | SC4 | g08Opr | 8 | 189.38 | 6.205 | 4.342 | 0.883  (1.012) | -0.853  (1.079) | -1.637  (3.353) | 3:1 |
| 41 | TSH | HBE140 | 8 | 215.73 | 3.612 | 4.101 | -2.434  (2.914) | -1.459  (0.649) | -0.086  (0.002) | 1:1 |

**Table S5.** Characteristics of the 111 QTLs mapped for all phenotypic traits: *R²*, LOD score, additive and dominance effects, segregation type.

| QTL | Trait | Flanking Markers | Linkage Group (LG) | Position  in cM | Global LOD | *R^2^* | α_PR 255_  (LOD) | α_PB 217_  (LOD) | δ_PR 255 x PB 217_  (LOD) | Segregation |
| --- | --- | --- | --- | --- | --- | --- | --- | --- | --- | --- |
| 41 | SC2 | HBE140 – g08T2587 | 8 | 219.00 | 3.625 | 3.941 | -0.545  (0.956) | -0.782  (1.469) | -0.693  (0.979) | 3:1 |
|  | SC3 | HBE140 – g08T2587 | 8 | 219.00 | 6.848 | 7.267 | 0.431  (0.301) | -1.888  (4.226) | -1.037  (1.194) | 1:2:1 |
| 42 | TSC | g08T2587 | 8 | 235.01 | 6.361 | 5.525 | 1.568  (0.537) | -5.927  (3.138) | -4.467  (2.109) | 1:2:1 |
| 43 | WC4 | g09TAs2547 – g09A2682 | 9 | 30.00 | 3.578 | 1.321 | -0.870  (2.202) | 0.350  (1.230) | -0.109  (0.427) | 1:1:1:1 |
| 44 | SH2 | g09A2682 | 9 | 39.08 | 4.794 | 3.655 | -1.542  (2.526) | 1.520  (2.387) | 0.059  (0.004) | 1:2:1 |
| 45 | SH1 | HBE77 | 9 | 99.63 | 4.171 | 1.297 | -1.618  (3.659) | 1.099  (0.735) | -1.679  (1.061) | 1:2:1 |
| 46 | TLP | EHBp21 | 9 | 109.04 | 4.768 | 2.614 | -21.560  (4.768) | – | – | 1:1 |
| 47 | SC1 | g10a129 | 10 | 0.00 | 3.623 | 6.449 | 0.579  (2.006) | -0.881  (1.811) | 0.377  (0.376) | 1:2:1 |

**Table S5.** Characteristics of the 111 QTLs mapped for all phenotypic traits: *R²*, LOD score, additive and dominance effects, segregation type.

| QTL | Trait | Flanking Markers | Linkage Group (LG) | Position  in cM | Global LOD | *R^2^* | α_PR 255_  (LOD) | α_PB 217_  (LOD) | δ_PR 255 x PB 217_  (LOD) | Segregation |
| --- | --- | --- | --- | --- | --- | --- | --- | --- | --- | --- |
| 48 | TWC | A2767 | 10 | 27.30 | 3.406 | 1.912 | 0.393  (0.285) | -0.704  (0.816) | -1.327  (2.951) | 1:1 |
| 49 | WC4 | g10A2438 – HBE204 | 10 | 123.00 | 3.867 | 2.987 | -0.066  (0.146) | -0.194  (1.280) | 0.279  (2.433) | 1:2:1 |
| 50 | WC3 | HB135 | 10 | 200.40 | 4.211 | 2.629 | -0.115  (1.162) | -0.134  (1.336) | 0.154  (2.252) | 3:1 |
| 51 | WH2 | HB186 | 10 | 215.90 | 4.500 | 4.832 | -0.861  (3.183) | 0.174  (0.133) | 0.470  (0.716) | 1:1 |
| 52 | WC2 | EHBc46 – HB170 | 10 | 262.00 | 7.710 | 0.589 | 0.823  (3.743) | 0.644  (3.136) | 0.337  (1.059) | 1:1:1:1 |
|  | WC2 | EHBc46 – HB170 | 10 | 263.00 | 7.755 | 0.504 | 0.833  (3.847) | 0.656  (3.188) | 0.353  (1.154) | 3:1 |
| 53 | WH1 | sHbUNI0518_S | 10 | 302.22 | 5.735 | 4.510 | -1.762  (4.080) | 0.795  (0.874) | -1.631  (3.385) | 3:1 |
| 54 | TSC | TAs2173 – g11A2535 | 11 | 87.00 | 3.993 | 2.990 | 1.171  (0.270) | 4.570  (3.768) | 1.685  (0.505) | 1:1 |

**Table S5.** Characteristics of the 111 QTLs mapped for all phenotypic traits: *R²*, LOD score, additive and dominance effects, segregation type.

| QTL | Trait | Flanking Markers | Linkage Group (LG) | Position  in cM | Global LOD | *R^2^* | α_PR 255_  (LOD) | α_PB 217_  (LOD) | δ_PR 255 x PB 217_  (LOD) | Segregation |
| --- | --- | --- | --- | --- | --- | --- | --- | --- | --- | --- |
| 54 | WC2 | TAs2173 – g11A2535 | 11 | 87.00 | 7.106 | 0.257 | 0.434  (1.586) | 0.775  (4.653) | 0.620  (2.934) | 3:1 |
|  | WC2 | TAs2173 – g11A2535 | 11 | 88.00 | 7.107 | 0.200 | 0.441  (1.594) | 0.784  (4.653) | 0.631  (2.980) | 3:1 |
| 55 | WC3 | EHBle3 – Hb_seq_197 | 11 | 141.00 | 4.060 | 3.859 | 0.178  (2.146) | 0.154  (2.065) | 0.073  (0.368) | 1:2:1 |
| 56 | SH1 | MnSOD | 11 | 174.08 | 8.063 | 6.479 | -1.390  (3.237) | -2.207  (5.511) | -0.609  (0.585) | 1:2:1 |
| 57 | TSH | HB_seq_29 | 12A | 36.28 | 4.205 | 2.485 | -1.809  (1.658) | 2.243  (2.603) | -0.604  (0.195) | 1:2:1 |
| 58 | WC3 | 512B5 | 13 | 140.77 | 3.416 | 3.223 | -0.200  (3.269) | 0.058  (0.117) | -0.036  (0.044) | 1:1 |
| 59 | TWH | g13A2757 – HBE98 | 13 | 174.00 | 4.368 | 4.625 | 1.474  (1.549) | 0.682  (0.349) | 1.994  (2.945) | 1:2:1 |
| 60 | WC3 | HBE156 | 14 | 30.16 | 7.451 | 1.078 | -0.230  (4.659) | -0.153  (2.124) | -0.065  (0.358) | 1:2:1 |

**Table S5.** Characteristics of the 111 QTLs mapped for all phenotypic traits: *R²*, LOD score, additive and dominance effects, segregation type.

| QTL | Trait | Flanking Markers | Linkage Group (LG) | Position  in cM | Global LOD | *R^2^* | α_PR 255_  (LOD) | α_PB 217_  (LOD) | δ_PR 255 x PB 217_  (LOD) | Segregation |
| --- | --- | --- | --- | --- | --- | --- | --- | --- | --- | --- |
| 60 | WC3 | sHbCIR0076 | 14 | 30.30 | 7.419 | 1.054 | -0.230  (4.626) | -0.152  (2.121) | -0.066  (0.369) | 1:2:1 |
|  | SC3 | sHbCIR0268 – HB53 | 14 | 33.00 | 5.557 | 4.395 | -1.387  (3.624) | -0.555  (0.569) | 0.999  (1.629) | 1:2:1 |
| 61 | TLP | Hb_seq_187 – HB100 | 14 | 52.00 | 5.191 | 2.538 | -19.791  (4.110) | 6.118  (0.398) | 12.982  (1.575) | 1:2:1 |
| 62 | SH1 | EHBc99 | 14 | 75.83 | 4.352 | 3.989 | -0.312  (0.145) | -0.664  (0.670) | -1.552  (3.314) | 1:1 |
| 63 | WC4 | sHbCIR0079 – EHBc33 | 14 | 137.00 | 6.152 | 4.469 | 0.200  (1.293) | 0.352  (4.120) | -0.203  (1.262) | 3:1 |
| 64 | TWH | EHBc33 – sHbCIR0273 | 14 | 152.00 | 4.894 | 1.333 | -3.023  (2.589) | -0.645  (0.319) | 1.724  (1.768) | 1:2:1 |
| 65 | WH2 | g15TA2163 – HB81 | 15 | 44.00 | 4.594 | 2.943 | -0.327  (0.452) | 0.441  (0.806) | 0.994  (3.951) | 1:1 |
| 66 | SC5 | g15TAs2706 – HBE10 | 15 | 74.00 | 3.398 | 2.219 | -0.869  (3.059) | 0.181  (0.141) | 0.227  (0.191) | 1:1 |

**Table S5.** Characteristics of the 111 QTLs mapped for all phenotypic traits: *R²*, LOD score, additive and dominance effects, segregation type.

| QTL | Trait | Flanking Markers | Linkage Group (LG) | Position  in cM | Global LOD | *R^2^* | α_PR 255_  (LOD) | α_PB 217_  (LOD) | δ_PR 255 x PB 217_  (LOD) | Segregation |
| --- | --- | --- | --- | --- | --- | --- | --- | --- | --- | --- |
| 66 | WC4 | g15TAs2706 – HBE10 | 15 | 75.00 | 3.505 | 4.506 | -0.144  (0.633) | 0.022  (0.018) | 0.323  (3.117) | 1:1 |
| 67 | WC3 | HB_seq_02_1 | 15 | 90.88 | 6.559 | 5.988 | -0.226  (4.350) | 0.089  (0.712) | -0.105  (1.022) | 1:2:1 |
| 68 | TWC | HB_seq_02_1 – sHbUNI0327 | 15 | 97.00 | 6.842 | 6.746 | -1.833  (5.119) | 0.740  (0.574) | 0.424  (0.239) | 1:1 |
|  | WC2 | sHbUNI0327 | 15 | 100.28 | 4.413 | 5.550 | -0.745  (4.030) | 0.190  (0.234) | -0.049  (0.016) | 1:1 |
| 69 | SC4 | HBE192 – EHBc8 | 15 | 127.00 | 3.579 | 3.448 | -1.400  (2.166) | 0.015  (0.002) | -1.049  (1.112) | 1:2:1 |
| 70 | TSC | EHBc8 | 15 | 139.66 | 3.494 | 1.358 | -2.733  (1.745) | 3.981  (1.754) | 0.434  (0.045) | 1:2:1 |
| 71 | SH2 | g15A2697 – g15A2492 | 15 | 147.00 | 3.618 | 2.760 | -0.962  (0.870) | -1.840  (3.042) | 0.500  (0.219) | 1:2:1 |
| 72 | SC2 | HBE64 | 16 | 0.00 | 6.720 | 3.375 | 0.039  (0.005) | 1.792  (5.306) | 0.690  (1.719) | 1:1:1:1 |

**Table S5.** Characteristics of the 111 QTLs mapped for all phenotypic traits: *R²*, LOD score, additive and dominance effects, segregation type.

| QTL | Trait | Flanking Markers | Linkage Group (LG) | Position  in cM | Global LOD | *R^2^* | α_PR 255_  (LOD) | α_PB 217_  (LOD) | δ_PR 255 x PB 217_  (LOD) | Segregation |
| --- | --- | --- | --- | --- | --- | --- | --- | --- | --- | --- |
| 72 | TSC | HBE64 – HBE84 | 16 | 4.00 | 5.137 | 3.932 | -1.531  (0.420) | 4.254  (3.789) | 1.722  (0.515) | 1:1 |
|  | TLP | HBE64 – HBE84 | 16 | 6.00 | 12.747 | 9.184 | -3.824  (0.163) | -32.267  (10.682) | -18.152  (3.223) | 1:1:1:1 |
| 73 | SC5 | HBE84 | 16 | 11.76 | 6.242 | 6.328 | 0.032  (0.005) | 1.135  (5.931) | 0.218  (0.218) | 1:1 |
| 74 | SC1 | g16a58 | 16 | 33.42 | 4.513 | 4.344 | -0.476  (1.593) | -0.620  (1.056) | 0.551  (2.159) | 3:1 |
| 75 | SC4 | g16a58 – g16t97 | 16 | 45.00 | 3.776 | 1.436 | -0.977  (1.315) | 0.621  (0.586) | 1.326  (2.525) | 1:2:1 |
|  | SC3 | g16t97 | 16 | 46.01 | 4.356 | 4.123 | -1.237  (2.783) | 0.109  (0.022) | 1.091  (2.186) | 1:2:1 |
| 76 | WC3 | EHBc97 – g17a78 | 17 | 14.00 | 4.009 | 3.361 | 0.110  (1.018) | -0.103  (0.750) | -0.199  (2.437) | 1:2:1 |
| 77 | SC3 | sHbUNI0517 | 17 | 72.63 | 6.071 | 4.157 | 1.019  (1.873) | -0.714  (0.878) | 2.257  (2.542) | 1:1:1:1 |

**Table S5.** Characteristics of the 111 QTLs mapped for all phenotypic traits: *R²*, LOD score, additive and dominance effects, segregation type.

| QTL | Trait | Flanking Markers | Linkage Group (LG) | Position  in cM | Global LOD | *R^2^* | α_PR 255_  (LOD) | α_PB 217_  (LOD) | δ_PR 255 x PB 217_  (LOD) | Segregation |
| --- | --- | --- | --- | --- | --- | --- | --- | --- | --- | --- |
| 78 | TLP | Hb_seq_195 – Hb_seq_163 | 17 | 86.00 | 4.950 | 3.700 | 18.471  (3.425) | 15.336  (2.246) | -3.992  (0.153) | 1:2:1 |
| 79 | TSC | g18T2113 | 18A | 19.54 | 6.781 | 3.820 | -2.674  (1.696) | 0.438  (0.046) | 6.672  (4.782) | 1:1:1:1 |
| 80 | SC2 | Hb_seq_24 – HB49 | 18A | 27.00 | 3.684 | 2.437 | -0.393  (0.555) | 0.513  (0.923) | 0.788  (2.060) | 1:2:1 |
| 81 | SC5 | HB49 – HB15 | 18A | 33.00 | 3.808 | 5.534 | -0.855  (3.425) | -0.277  (0.358) | 0.218  (0.220) | 1:1 |
| 82 | TLP | Hb_seq_107 | 18A | 97.90 | 7.454 | 2.028 | 7.982  (0.878) | 69.677  (5.149) | -9.150  (1.311) | 1:2:1 |
| 83 | TLP | g10a288 | 18B | 4.02 | 6.310 | 4.150 | 1.445  (0.022) | -12.939  (1.679) | -19.952  (4.329) | 1:2:1 |
|  | WH2 | TAs2744_1 | 18B | 8.34 | 4.141 | 2.564 | 0.861  (3.404) | -0.471  (0.845) | -0.048  (0.009) | 1:2:1 |
